# Sustainable agility of product development process based on a rough cloud technique: A case study on China’s small and medium enterprises

**DOI:** 10.1371/journal.pone.0300266

**Published:** 2024-08-22

**Authors:** Zhining Zhao, Hassan Alli, Masoud Ahmadipour, Rosalam Che me

**Affiliations:** 1 Faculty of Design and Architecture, Universiti Putra Malaysia, UPM, Serdang, Selangor, Malaysia; 2 Faculty of Fine Art and Design, Qiqihar University, Qiqihar City, Heilongjing, Province China; 3 Department of Electrical and Electronics Engineering, Institute of Power Engineering, College of Engineering, Universiti Tenaga Nasional (UNITEN), Kajang, Selangor, Malaysia; Industrial University of Ho Chi Minh City, VIET NAM

## Abstract

The importance of incorporating an agile approach into creating sustainable products has been widely discussed. This approach can enhance innovation integration, improve adaptability to changing development circumstances, and increase the efficiency and quality of the product development process. While many agile methods have originated in the software development context and have been formulated based on successful software projects, they often fail due to incorrect procedures and a lack of acceptance, preventing deep integration into the process. Additionally, decision-making for market evaluation is often hindered by unclear and subjective information. Therefore, this study introduces an extended TOPSIS (Technique for Order Performance by Similarity to Ideal Solution) method for sustainable product development. This method leverages the benefits of cloud model theory to address randomness and uncertainty (intrapersonal uncertainty) and the advantages of rough set theory to flexibly handle market demand uncertainty without requiring extra information. The study proposes an integrated weighting method that considers both subjective and objective weights to determine comprehensive criteria weights. It also presents a new framework, named Sustainable Agility of Product Development (SAPD), which aims to evaluate criteria for assessing sustainable product development. To validate the effectiveness of this proposed method, a case study is conducted on small and medium enterprises in China. The obtained results show that the company needs to conduct product structure research and development to realize new product functions.

## 1. Introduction

Sustainable design, also referred to as design for sustainability or sustainable product development, seeks to revolutionize product development practices with the overarching goal of ensuring the flourishing of all species for generations to come [1–5]. This concept of sustainable design has evolved from principles found in green design, ecological design, and life cycle design [6]. It transcends the narrow focus on environmental protection and resource conservation, extending its reach to encompass the sustainable advancement of human well-being, society, culture, and the economy. Its overarching aim is to establish a balanced and harmonious relationship among people, the environment, and society. Moreover, contemporary sustainable development requirements in product design also emphasize the incorporation of social culture and social innovation, along with services and systems integration into the design process. Typically, product design is carried out within the Research and Development (R&D) department, where designers, engineers, and, in some instances, scientists collaborate [7,8]. As the concept of sustainable design has continued to evolve, it has emerged as a strategic approach for creating and advancing sustainable solutions [9]. By implementing systematic planning for the entire product during the product development phase, designers can infuse sustainability throughout the entire product life cycle [10]. This evolving perspective on sustainable design not only opens more opportunities for success in the market but also presents fresh challenges to product designers. The ability to continuously process and convert new information into tangible products has become a fundamental requirement for product designers [11]. Simultaneously, in response to the dynamic market environment, small and medium-sized enterprises in China are actively seeking faster and more efficient methods to enhance the value of their products [12]. Agile management offers companies the capacity to swiftly address customer needs and deliver high-quality products, thereby bolstering their competitiveness in the market [13]. When enterprises embark on sustainable product development, agile management streamlines the development process and aids in making resource allocation decisions that align with time and efficiency demands. This not only enables companies to adhere to development schedules but also mitigates the risks associated with changes in the process.

Agility in the context of sustainable product development can be viewed as a multi-criteria decision-making process [14–16]. Selecting an appropriate agility management method is crucial for helping a company maximize the benefits of sustainable product development. Currently, many studies have demonstrated the significant impact of the choice of design method on the product’s entire life cycle [17,18]. While these studies have primarily concentrated on supplier selection under supply chain practices, there’s a notable absence of research focusing on supplier selection encompassing economic, environmental, and social dimensions. This emphasizes the need for further exploration and development in this area to address the broader spectrum of sustainability concerns within sustainable design methods. However, it is worth noting that previous research has indicated that the selection of sustainable design methods in product development often involves uncertain information, which necessitates the use of cloud models and fuzzy set theory to capture this ambiguity [19,20]. Moreover, there are limitations associated with the application of fuzzy set theory in Multi-Criteria Decision Making (MCDM) processes for sustainable design methods. These drawbacks include exact membership degrees that fail to address vagueness adequately, reliance on pre-set membership functions which may not accurately represent real-world uncertainties, and a lack of consideration for interpersonal uncertainties among decision-makers [19]. These limitations underscore the necessity for alternative or complementary approaches to enhance the robustness and effectiveness of MCDM techniques in supplier selection processes [20]. To ensure the accuracy of decision-making, both subjective and objective weights are taken into consideration to determine the combined weights [21]. Considering these factors, the primary objective of this study is to build upon previous methods to enhance the accuracy of practices and outcomes by introducing a SAPD process. This method seeks to evaluate sustainability criteria for products more effectively.

In this design approach, the SAPD is structured in several steps. First, SAPD practices are defined as the benchmark for market evaluation. Second, a prioritization model for sustainability and agility design methods is introduced, capable of managing various uncertainties. This model integrates cloud model theory to handle personal information uncertainty and rough set theory to address market demand uncertainty. Third, a combined weighting technique is developed to calculate the weight of SAPD practices, ensuring a comprehensive reflection of the standards’ relative importance. Finally, to demonstrate the viability of this method, it was applied to a specific case: the design of a shower for a Chinese sanitary ware company.

## 2. Background of study

### 2.1. Sustainable design methods

In the recent landscape of sustainable product development, there has been a greater emphasis on sustainability as a corporate development strategy, with less attention given to the specific design methods that companies can implement. There is a growing need to enhance guidelines that facilitate the gradual transition towards sustainable development by encouraging companies to embrace alternative sustainable solutions [22]. This involves the incorporation of systems thinking and life cycle analysis to infuse sustainability considerations across the entire product life cycle during the development planning phase [23]. Design is also seen as a powerful tool for influencing and shaping customer needs and behaviors to drive sustainable development initiatives [24]. Several studies suggest that the application of systematic frameworks plays a pivotal role in sustainable design. They propose that the key to achieving sustainable design lies in the integration of economic, environmental, and social considerations [25,26]. Moreover, they highlight that the success of sustainable practices requires solid theoretical foundations and standardized intervention measures to guide these practices effectively [27]. In this context, models like the driver and barrier model of sustainable design have been established to promote sustainable design by reinforcing drivers and mitigating barriers through a systematic approach [28]. To consolidate these insights, a review of research on sustainable design has resulted in the identification of commonly used sustainable design methods, as summarized in Table A.1 in S1 File.

With the aid of multiple criteria and various design tools, sustainable design evolves into a decision-making process that considers multiple criteria. In practical operations, companies often encounter product development challenges with uncertain or imprecise information [29]. To address this issue, scholars propose the use of fuzzy set theory to choose more suitable design methods, helping companies mitigate the influence of uncertain and imprecise factors in product development decisions. For instance, they adopt the concept of quality function deployment (QFD) to model customer and technical requirements and establish the relationship between them. They then employ a priority matrix to prioritize customer requirements and use gap analysis to screen technical requirements. QFD is utilized to determine requirement weights, providing decision-makers with a structured approach for selecting areas for improvement [30]. Ranking preferences involve employing the TOPSIS to assess performance by considering the distance and similarity between alternatives [27]. In a similar vein, it begins with a comprehensive evaluation of the entire component life cycle (LC) and compares various design solutions using the TOPSIS method [31]. Primary and secondary design criteria are established based on customer needs, and fuzzy technology is utilized to assess the design criteria for preference ordering. Optimal solutions are determined through multi-objective and single-objective binary programming models, thereby providing decision makers with both multi-objective and single-objective optimal solutions [32].

### 2.2. Agility management method

Several companies are consistently enhancing their sustainable development capabilities, while simultaneously grappling with intense market competition and ever-changing customer demands. To address these challenges, enterprises must possess agility capabilities [33–35]. Agile capabilities denote a company’s aptitude for identifying shifts in its business environment and swiftly reorganizing its resources, procedures, and strategies to promptly address these changes. This capacity fosters collaboration within the enterprise and centers around knowledge, personnel, technology, and processes, enabling companies to adapt to shifts and capitalize on development opportunities in a dynamic market environment [36].

The four dimensions of agility encompass 1) delivering value to customers, 2) preparing for change, 3) valuing human knowledge and skills, and 4) building virtual partnerships [37]. Enterprises equipped with agile capabilities can efficiently manage the production and promotion of sustainable products, enabling them to gain a strong foothold in market competition. Furthermore, these agility capabilities assist enterprises in managing internal and external relationships from a managerial standpoint, fostering a favorable production environment [38].

Scholars from various disciplines have researched agility. Some studies have highlighted the importance of identifying appropriate agile capabilities during the strategic goal-setting stage and have introduced systematic approaches for identifying and ranking agility indicators [39,40]. Others have emphasized the strategic sensitivity and resourcefulness of agility capabilities in helping enterprises surmount challenges [41,42]. Furthermore, some studies have explored the connection between agility capabilities and manufacturing. For instance, these capabilities enhance flexibility in product life cycle management, enabling better alignment with the evolving requirements of an enterprise’s overall product planning [43]. Enhancing a company’s agility can enhance the speed, efficiency, and quality of manufacturing processes, significantly influencing the performance of small and medium-sized businesses [44]. Agility capabilities enable businesses to pinpoint negative aspects in their manufacturing procedures, empowering managers to uphold and enhance organizational flexibility [45]. Regarding design, team management’s agility capability enables businesses to create a seamless communication platform, facilitating efficient product development and adaptation to market changes [46]. Refer to Table A.2 in S1 File for various agile management approaches employed by enterprises. Agility in enterprises manifests itself in various dimensions, including corporate strategy, manufacturing technology, and management systems [39]. Consequently, fostering agility entails the active involvement of enterprise managers in decision-making and ongoing enhancement. Numerous studies have contributed to the enhancement of enterprise agility, encompassing aspects like agile manufacturing and design agility [47,48]. Nevertheless, the selection of agility methods is merely one aspect of achieving agility. Approaches such as data collection, cluster analysis, or group interviews are often used to make informed decisions regarding agile practices. However, there is a limited body of peer-reviewed literature that scrutinizes the selection of agility methods through a more objective lens.

### 2.3. Integration of sustainable design methods and agile management methods

Considering substantial pressures related to resources, the environment, and market dynamics, businesses are actively in pursuit of product development approaches that can address both sustainability demands and market fluctuations concurrently [38]. The fusion of sustainable design methodologies with agile management techniques brings together environmentally conscious product development practices and versatile, adaptable project management strategies. This harmonious combination empowers organizations to incorporate sustainability factors seamlessly and efficiently into their product development processes. This, in turn, helps minimize environmental and social impacts while enhancing their ability to respond to evolving market demands and opportunities. Academics have put forth various approaches to attain both agility and sustainability in product development simultaneously. Certain studies emphasize that agile management can effectively manage uncertainties and unforeseen alterations across the product life cycle. It fosters enhanced collaboration within design teams, integrates customers into the development process, encourages knowledge reuse, and facilitates the swift configuration of products and processes [49]. In addition, some scholars suggest that agile management contributes to the advancement of enterprise information technology and enhances the likelihood of success in sustainable product design by translating customer requirements into well-defined technical solutions [50]. Additionally, agile management emphasizes that cross-functional teams should engage in information sharing and concurrent engineering by leveraging cloud computing, big data, and other intelligent technologies [51]. This facilitates the deliberate integration of sustainable design principles, life cycle assessments, product adaptability, and the implementation of the Cradle-to-Cradle design concept. Furthermore, some scholars have highlighted the benefits of modular design in both sustainability and agility [52,53]. A thoughtfully designed modular product architecture streamlines the standardization and generalization of components, thereby influencing the product’s entire life cycle.

Simultaneously, modular product components contribute to time savings when it comes to product modifications and upgrades, reflecting agility. While current research has underscored the advantages that the fusion of agile management and sustainable design can offer to enterprises, these studies have presented various viewpoints and assessed the effectiveness of these methods without addressing their widespread adoption. In many theoretical studies, businesses find it challenging to determine development strategies that align with their specific needs for both agility and sustainability. To bridge this gap, this study explores the use of the rough cloud model to translate sustainable methods and agile management techniques into data sets. In this respect, TOPSIS has been utilized frequently in the field of sustainable product development. TOPSIS stands for "Technique for Order of Preference by Similarity to Ideal Solution," which is a technique within Multiple Criteria Decision Making (MCDM) used to identify the best solution among a limited set of alternatives. It offers several benefits: (1) it generates a single value that considers both the best and worst options concurrently; (2) it follows a logical approach that mirrors human decision-making; (3) it allows the visualization of performance measures for all alternatives across attributes on a polyhedron, especially in cases involving two dimensions at least; and (4) it involves a straightforward computational process that can be easily implemented in spreadsheet programs [54]. These advantages establish TOPSIS as a prominent method within Multiple Criteria Decision Making (MCDM), distinguishing it from other comparable methods like AHP and ELECTRE [55]. However, the conventional TOPSIS approach cannot handle the inherent uncertain and imprecise information found in expert evaluations. Fuzzy-based variations of TOPSIS are frequently employed to enhance the supplier selection process [56]. Nevertheless, there hasn’t been any research combining cloud model theory and rough set theory within TOPSIS to manage uncertainties. In this regard, this paper introduces a novel TOPSIS methodology designed for sustainable product development. This approach aims to furnish companies with more rational and well-informed decision-making solutions.

The primary contributions of this paper can be summarized as follows:

- The identification of Sustainable Agility of Product Development (SAPD) as key evaluation criteria.
- Addressing uncertainty stemming from randomness and fuzziness through the application of cloud model theory.
- Introducing rough numbers to manage interpersonal uncertainty during decision-making processes.
- Utilizing an integrated weighting method to ascertain the importance of selection criteria.
- Validating the effectiveness of the method through its application in small and medium enterprises based in China.

## 3. Material and method

### 3.1 Cloud model

The cloud model theory, stemming from both fuzzy set theory and probability theory, is an artificial intelligence approach capable of representing knowledge that involves uncertainty and randomness [57]. Cloud model theory incorporates random variations around a specified central membership value and interprets membership as a precise quantity. In the context of cloud model theory, when considering *T* as a qualitative concept defined within the domain of discourse denoted as *U*, any element *x* within the domain of discourse *U* can be examined. In this framework, the membership degree of *x* to the concept *T*, represented as *μT*(*x*), exists within the interval [0,1]. It’s important to note that *μT*(*x*) is not a constant, but rather a stochastic variable conforming to a probability distribution. Within this context, the arrangement of elements within the domain *U* is referred to as a "cloud," and each specific element, like *x*, is termed *a* "cloud droplet."

In the cloud model, three quantitative parameters are employed to describe the distribution of elements: expectation (*Ex*), entropy (*En*) and superentropy (*He*). Expectation (*Ex*) denotes the mathematical average value of the cloud droplet and holds significant importance as it encapsulates the core essence of the qualitative concept. Entropy (*En*) serves as an indicator of the randomness and ambiguity inherent in qualitative concepts. It gauges both the dispersion of cloud droplets and the range of the universe covered by the concept. In essence, Entropy (*En*) quantifies the levels of uncertainty and variability within a given concept. Hyper-entropy (*He*) is derived from the entropy En and serves as a measure of the uncertainty linked to membership. In this framework, clouds can be succinctly expressed as *C* = (*Ex*,*En*,*He*), with these three parameters collectively offering a comprehensive depiction of the underlying distribution of elements within the qualitative concept.

The standard cloud model finds extensive applications and can effectively represent various uncertain phenomena. Within the normal cloud model, for a qualitative concept *T*, a random instantiation of *x* is described by the following equation:

$$x∼N\left(Ex,{En^{\prime }}^{2}\right)$$ (1)

$$En^{\prime }∼N\left(En,He^{2}\right)$$ (2)

The degree of membership of *x* in the qualitative concept *T* complies with the following equation:

$$\mu_{T}(x)=e^{\frac{{\left(x-Ex\right)}^{2}}{2{\left(En^{\prime }\right)}^{2}}}$$ (3)

When the expected value Ex falls within the interval $\left[\underline{Ex},\bar{Ex}\right]$, the cloud model transforms into an interval cloud, denoted as $\tilde{C}=([\underline{Ex},\bar{Ex}],En,He)$.

**Definition 1.** There exist two interval clouds [58]:

${\tilde{C}}_{1}=\left(\left[\underline{Ex_{1}},\bar{Ex_{1}}\right],En_{1},He_{1}\right)$ and ${\tilde{C}}_{2}=\left(\left[\underline{Ex_{2}},\bar{Ex_{2}}\right],En_{2},He_{2}\right)$. The rules for arithmetic operations are presented as follows:

$${\tilde{C}}_{1}+{\tilde{C}}_{2}=\left(\left[\underline{Ex_{1}}+\underline{Ex_{2}},\bar{Ex_{1}}+\bar{Ex_{2}}\right],\sqrt{En_{1}^{2}+En_{2}^{2}},\sqrt{He_{1}^{2}+He_{2}^{2}}\right)$$ (4)

$${\tilde{C}}_{1}\times {\tilde{C}}_{2}=\left(\underline{Ex_{1}Ex_{2}},\bar{Ex_{1}Ex_{2}},\sqrt{{\left(En_{1}Ex_{2}\right)}^{2}+{\left(En_{2}Ex_{1}\right)}^{2}},\sqrt{{\left(He_{1}Ex_{2}\right)}^{2}+{\left(He_{2}Ex_{1}\right)}^{2}}\right)$$ (5)

$${\tilde{C}}_{1}^{m}=\left(\left\lceil \underline{Ex_{1}^{m}},\bar{Ex_{1}^{m}}\right\rceil ,,,\sqrt{m},\times ,E,x_{1}^{m-1},\times ,E,n_{1},,,\sqrt{m},\times ,E,x_{1}^{m-1},\times ,H,e_{1}\right)$$ (6)

$$\lambda {\tilde{C}}_{1}=\left(\left\lceil \lambda \underline{Ex_{1}},\lambda \bar{Ex_{1}}\right\rceil ,\sqrt{\lambda }En_{1},\sqrt{\lambda }He_{1}\right)$$ (7)

Where *λ* is a constant satisfying λ > 0. $Ex_{1}=\left(\underline{Ex_{1}}+\bar{Ex_{1}}\right)/2$ and $Ex_{2}=\left(\underline{Ex_{2}}+\bar{Ex_{2}}\right)/2$.

**Definition 2.** Let ${\tilde{C}}_{1}=\left(\left[\underline{Ex_{1}},\bar{Ex_{1}}\right],En_{1},He_{1}\right)$ and ${\tilde{C}}_{2}=\left(\left[\underline{Ex_{2}},\bar{Ex_{2}}\right],En_{2},He_{2}\right)$ be two arbitrary interval clouds in the domain *U*. The definition of the distance between the two interval clouds is as follows: [59]:

$$\begin{array}{l} d\left({\tilde{C}}_{1},{\tilde{C}}_{2}\right)=\frac{1}{2}\times \left(\left|\left(1-\frac{En_{1}+He_{1}}{Ex_{1}}\right)\underline{Ex_{1}}-\left(1-\frac{En_{2}+He_{2}}{Ex_{2}}\right)\underline{Ex_{2}}\right|\right.\\ \left.\;+\left|\left(1-\frac{En_{1}+He_{1}}{Ex_{1}}\right)\bar{Ex_{1}}-\left(1-\frac{En_{2}+He_{2}}{Ex_{2}}\right)\bar{Ex_{2}}\right|\right) \end{array}$$ (8)

Where $d\left({\tilde{C}}_{1},{\tilde{C}}_{2}\right)$ is the distance between the two interval clouds ${\tilde{C}}_{1}$ and ${\tilde{C}}_{2}$. $Ex_{1}=\left(\underline{Ex_{1}}+\bar{Ex_{1}}\right)/2$ and $Ex_{2}=\left(\underline{Ex_{2}}+\bar{Ex_{2}}\right)/2$.

**Definition 3:** Consider two interval clouds ${\tilde{C}}_{1}=\left(\left[\underline{Ex_{1}},\bar{Ex_{1}}\right],En_{1},He_{1}\right)$ and ${\tilde{C}}_{2}=\left(\left[\underline{Ex_{2}},\bar{Ex_{2}}\right],En_{2},He_{2}\right)$ in the domain *U*.Based on the 3 *En* principle, the interval clouds can be transformed into intervals represented as $\alpha =[\underline{\alpha },\bar{\alpha }]$, and $b=[\underline{b},\bar{b}]$, where $\underline{\alpha }=\underline{Ex_{1}}-3En_{1}$, $\bar{\alpha }=\bar{Ex_{1}}+3En_{1},\underline{b}=Ex_{2}-3En_{2}$, and $\bar{b}=\bar{Ex_{2}}+3En_{2}$. Then, the two interval clouds can be compared based on the following ranking rules [60]:

1. If $R_{ab}>0,{\tilde{\text{C}}}_{1}>{\tilde{\text{C}}}_{2}$;
2. If $R_{ab}=0\;\text{and}\;En_{1}<En_{2}\;\text{then}\;{\tilde{\text{C}}}_{1}>{\tilde{\text{C}}}_{2};$
3. If $R_{ab}=0\;\text{and}\;En_{1}=En_{2},\;\text{and}\;He_{1}<He_{2},\text{then}\;{\tilde{\text{C}}}_{1}>\;{\tilde{\text{C}}}_{2};$
4. If $R_{ab}=0\;\text{and}\;En_{1}=En_{2},\;\text{and}\;He_{1}=He_{2},\text{then}\;{\tilde{\text{C}}}_{1}={\tilde{\text{C}}}_{2}.$

Where $R_{ab}=2(\bar{\alpha }-\underline{b})-(\bar{\alpha }-\underline{\alpha }+\bar{b}-\underline{b})$.

#### 3.1.1. Transformation between linguistic variables and cloud model

Linguistic variables are terms used to characterize qualitative attributes or traits that can represent intricate and ambiguously defined situations. To represent linguistic variables within a cloud model, it is necessary to assign them quantitative values.

**Definition 4.** The set of linguistic terms is typically finite and organized in a specific order, and it can be denoted as *S* = {*S*_*α*_ | α = 0,…,*t*, *t* ∈ N}. *S*_*α*_ represents a valid value within the linguistic term set named *S*, while *N* encompasses all non-negative integers. The characteristics of the linguistic term set *S* are as follows:

1. If *α* > *β*, then *S*_*α*_ > *S*_*β*_;
2. *Neg*(*S*_*a*_) = *S*_*β*_, where *β* = *t* − *α* and *Neg*(*S*_*α*_) is the negation operator.
3. If *S*_*α*_ > *S*_*β*_, then *max*{*S*_*α*_, *S*_*β*_} = *S*_*α*_, where *max*{*S*_*α*_, *S*_*β*_} is the max operator.

A hierarchical set of linguistic terms can typically be illustrated as depicted in Table A.3 in S1 File.

**Definition 5.** When the linguistic term set is aligned with the cloud model, and the linguistic term set is established as *S* = {*S*_*α*_ | α = 0,…,*t*, *t* ∈ N}, the fundamental clouds are derived as follows [61]:

$$C_{0}=\left(Ex_{0},En_{0},He_{0}\right),C_{1}=\left(Ex_{1},En_{1},He_{1}\right),\ldots ,C_{t}=\left(Ex_{t},En_{t},He_{t}\right)$$ (9)

When the numerical scale employed to express the significance of each indicator ranges between 0 and 1, the numeric distribution of the five levels along with their corresponding qualitative linguistic descriptions can be found in Table A.4 in S1 File [62].

Definition 6

When [*S*_*α*_, *S*_*β*_] signifies an interval value within the linguistic term set *S*, they can be converted into *C*_*α*_ = (*Ex*_*α*_, *En*_*α*_, *He*_*α*_) and *C*_*β*_ = (*Ex*_*β*_, *En*_*β*_, *He*_*β*_). Consequently, the corresponding interval cloud is as follows:

$$\tilde{C}=([\underline{Ex},\bar{Ex}],En,He)$$ (10)

$$\left\{\begin{array}{l} \underline{Ex}=min\left\{Ex_{\alpha },Ex_{\beta }\right\}\\ \bar{Ex}=max\left\{Ex_{\alpha },Ex_{\beta }\right\} \end{array}\right.$$ (11)

$$En=\sqrt{\left(En_{\alpha }^{2}+En_{\beta }^{2}\right)/2}$$ (12)

$$He=\sqrt{\left(He_{\alpha }^{2}+He_{\beta }^{2}\right)/2}$$ (13)

#### 3.1.2. Extended rough set-based cloud model

Rough set theory is another form of artificial intelligence that can represent the vagueness in the individual judgments of decision-makers within a group, and it does so without the need for additional information like membership functions, data distribution, or numerous fuzzy rules, which are commonly found in fuzzy set theory. In the context of rough set theory, decision-makers are required to make determinations. This approach can effectively capture the wide range of judgments made by decision-makers by employing flexible rough intervals rooted in clear assessments. It’s worth noting that when the rough interval is larger, it indicates a higher level of inconsistency among decision-makers [61]. Consequently, this theory finds application in addressing expert subjectivity in the selection of agility and sustainable design methods. However, it’s important to recognize that rough set theory is not designed to handle randomness.

Additionally, decision-makers frequently find it more convenient to use linguistic terms when responding to questions. It can be challenging for them to precisely convey their perceptions using numerical values. Consequently, this study combines the normal cloud model and rough set theory to address diverse sources of uncertainty, ultimately providing enterprises with more precise and objective evaluation results.

Let *IS* be the interval language judgment set provided by l experts, $IS=\left\{{\left.{\left[S_{\alpha },S_{\beta }\right]}_{i}\right|}_{\alpha },\beta =0,\ldots ,t,t\in N;i=1,2,\ldots ,l\right\}$. Subsequently, following definitions (5) and (6), the interval linguistic values can be transformed into interval cloud models. This leads to the representation of the interval cloud judgment set as follows:

$$ICS=\left\{{\tilde{C}}_{ı}=\left(\left|\underline{Ex_{i}},\bar{Ex_{i}}\right|\right),En_{i},He_{i}|i=1,2,\ldots ,l\right\}$$ (14)

Simultaneously, the lower approximation and upper approximation of each interval cloud can be calculated using the following equation:

$$\underline{Apr}\left({\tilde{C}}_{i}\right)=\cup \left\{{\tilde{C}}_{j}\in ICS|{\tilde{C}}_{j}\leq {\tilde{C}}_{i}\right\}$$ (15)

$$\bar{APr}\left({\tilde{C}}_{i}\right)=\text{u}\left\{{\tilde{C}}_{j}\in ICS|{\tilde{C}}_{j}\geq {\tilde{C}}_{i}\right\}$$ (16)

$\underline{Apr}\left({\tilde{C}}_{i}\right)$ and $\bar{APr}\left({\tilde{C}}_{i}\right)$ represent the lower approximation and upper approximation respectively. Then, the lower cloud limit and the upper cloud limit are defined as

$$\begin{array}{l} \underline{Lim}\left({\tilde{C}}_{i}\right)=\left(\left[Ex_{i}^{L},\bar{Ex_{i}^{L}}\right],En_{i}^{L},He_{i}^{L}\right)\\ \left.=\frac{1}{N_{l}}\sum_{j}{\tilde{C}}_{j}\right|{\tilde{C}}_{j}\in \underline{Apr}\left({\tilde{C}}_{i}\right)\\ =\left(\left[\frac{1}{N_{l}}\sum \underline{Ex_{j}},\frac{1}{N_{l}}\sum \bar{Ex_{j}}\right],\sqrt{\frac{1}{N_{l}}\sum {\left(En_{j}\right)}^{2}},\sqrt{\frac{1}{N_{l}}\sum {\left(He_{j}\right)}^{2}}\right) \end{array}$$ (17)

$$\begin{array}{c} \bar{Lim}\left({\tilde{C}}_{i}\right)=\left(\left[\underline{Ex_{i}^{U}},\bar{Ex_{i}^{U}}\right],En_{i}^{U},He_{i}^{U}\right)\\ \begin{array}{l} \left.=\frac{1}{N_{U}}\sum_{j}{\tilde{C}}_{j}\right|{\tilde{C}}_{j}\in \underline{Apr}\left({\tilde{C}}_{i}\right)\\ =\left(\left[\frac{1}{N_{u}}\sum \underline{Ex_{j}},\frac{1}{N_{U}}\sum \bar{Ex_{j}}\right],\sqrt{\frac{1}{N_{U}}\sum {\left(En_{j}\right)}^{2}},\sqrt{\frac{1}{N_{U}}\sum {\left(He_{j}\right)}^{2}}\right) \end{array} \end{array}$$ (18)

The rough cloud model can be elucidated as follows:

$$\begin{array}{c} R{\tilde{C}}_{i}=\left[\underline{Lim}\left(C_{i}\right),\bar{Lim}\left(C_{i}\right)\right]\\ =\left(\left(\left[\underline{Ex_{i}^{L}},\bar{Ex_{i}^{L}}\right],En_{i}^{L},He_{i}^{L}\right),\left(\left[\underline{Ex_{i}^{U}},\bar{Ex_{i}^{U}}\right],En_{i}^{U},He_{i}^{U}\right)\right) \end{array}$$ (19)

When the rough cloud model aligns with the basic cloud model,

$$\left.R{\tilde{C}}_{i}=([\underline{Ex_{i}^{\prime }},\bar{Ex_{i}^{\prime }}],En_{i}^{\prime },He_{i}^{\prime }\right)$$ (20)

Referring to definition (6), the rough cloud model is described as follows:

$$\left\{ \begin{array}{c} \underline{Ex_{i}^{'}}=min\left(\underline{Ex_{i}^{L}},\underline{Ex_{i}^{U}}\right)\\ \bar{Ex_{i}^{'}}=max\left(\bar{Ex_{i}^{L}},\bar{Ex_{i}^{u}}\right) \end{array}\right.$$ (21)

$$En_{i}^{\prime }=\sqrt{{\left(En_{i}^{L}\right)}^{2}+{\left(En_{i}^{U}\right)}^{2}/2}$$ (22)

$$He_{i}^{\prime }=\sqrt{{\left(He_{i}^{L}\right)}^{2}+{\left(He_{i}^{U}\right)}^{2}/2}$$ (23)

### 3.2. The proposed approach

**Fig 1:**
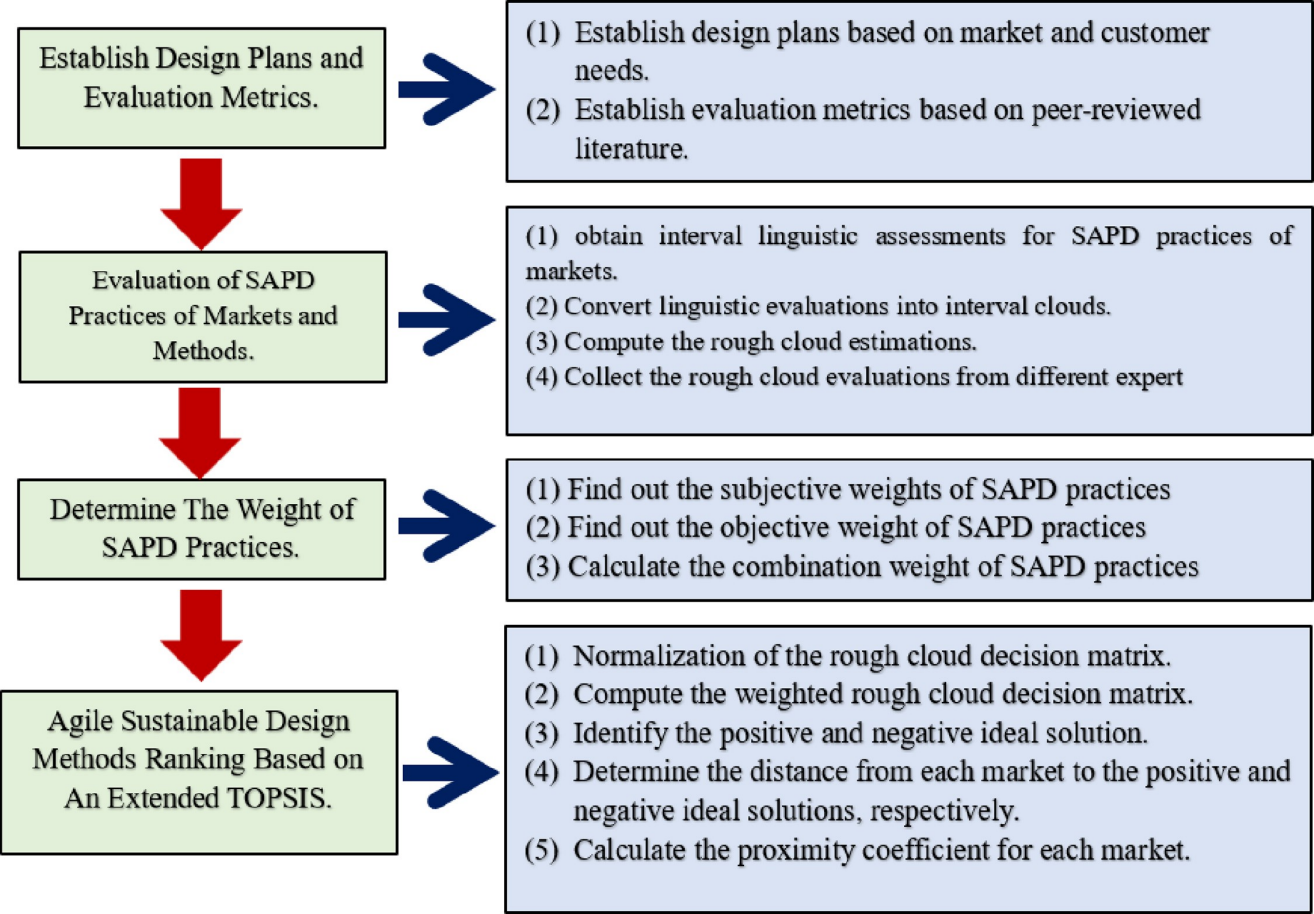
Flowchart illustration the proposed approach.

In this section, a hybrid model is created for evaluating SAPD practices in markets. This study integrates both cloud models and rough set theory to handle various sources of uncertainty. The flowchart illustrating the proposed method is presented in Fig 1.

#### 3.2.1. Establish design plans and evaluation metrics

Companies must create favorable design plans based on market and customer requirements. The description and scope of design plans that meet these requirements are qualitative and reflect the subjective judgment of the enterprise. Various design plans can be denoted as *A*1,*A*2,*A*3,…,*An*. When it comes to emphasizing agility and sustainability in design methods, specific indicators related to sustainable design methods and agile management were identified based on peer-reviewed literature. In different design plans, the emphasis on these indicators may vary.

#### 3.2.2. Evaluation of SAPD practices of markets, and product development method

To assess the SAPD practices in markets and product development methods, four key steps should be followed:

**Step 1:** Acquire the interval linguistic assessment for SAPD practices in markets and product development methods.

The choice of an agile sustainable design method depends on the experts’ evaluations of the relative importance of the methods. The linguistic term scales used for assessment are elaborated in Table A.5 in S1 File. Experts can contribute to decision-making in uncertain situations by providing a significant interval to express their judgment. The equation representing relative importance in interval linguistic terms is as follows:

$$F_{n}=\left(\left[f_{n}^{1L},f_{n}^{1U}\right],\left[f_{n}^{2L},f_{n}^{2U}\right],\ldots ,\left[f_{n}^{kL},f_{n}^{kU}\right]\right)$$ (24)

in which, *F*_*n*_ represents the interval linguistic judgment set for the importance of the *nth* SAPD practice’s importance provided by *l* experts. $\left[f_{n}^{kL},f_{n}^{kU}\right]$ denotes the interval linguistic preference given by the kth expert (*n* = 1,2,…,*j*; *k* = 1,2,…,*l*).

If we consider there are *m* agile sustainable design methods, the interval linguistic evaluation matrix appears as follows:

$$Y^{k}=\left[\begin{array}{cccc} \left[y_{11}^{kL},y_{11}^{kL}\right] & \left[y_{12}^{kL},y_{12}^{kU}\right] & \cdots & \left[y_{1n}^{kL},y_{1n}^{kU}\right]\\ \left[y_{21}^{kL},y_{21}^{kU}\right] & \left[y_{22}^{kL},y_{22}^{kU}\right] & \cdots & \left[y_{23}^{kL},y_{23}^{kU}\right]\\ \vdots & \vdots & \vdots & \vdots \\ \left[y_{m1}^{kL},y_{m1}^{kL}\right] & \left[y_{m2}^{kL},y_{m2}^{kL}\right] & \cdots & \left[y_{mn}^{kL},y_{mn}^{kL}\right] \end{array}\right]$$ (25)

In which, *Y*^*k*^ represents the matrix provided by *kth* experts $(k=1,2,\ldots ,l)\cdot \left[y_{in}^{kL},y_{in}^{kL}\right]$ denotes the interval linguistic preference given by the *kth* expert for the *ith* agile sustainable design method under the *nth* practice of agile management and sustainable design (i = 1, 2, …, m; n = 1, 2, …, j).

**Step 2:** Convert linguistic assessments into interval cloud.

Using the definitions (5) and (6), the linguistic assessments for the importance of SAPD practices can be transformed into interval clouds in the following manner:

$$\tilde{C}\left(\left[f_{n}^{kL},f_{n}^{kU}\right]\right)=\left(\left[\underline{Ex_{n}^{k}},{\bar{Ex}}_{n}^{k}\right],En_{n}^{k},He_{n}^{k}\right)$$ (26)

in which, $\tilde{C}\left(\left[f_{n}^{kL},f_{n}^{kU}\right]\right)$ is the interval cloud model of the linguistic importance $\left[f_{n}^{kL},f_{n}^{kU}\right]$.

Likewise, the individual linguistic assessments for product development plans under each SAPD practice can also be provided as Table A.5 in S1 File follows:

$$\tilde{C}\left(\left[y_{in}^{kL},y_{in}^{kU}\right]\right)=\left(\left[{\underline{Ex}}_{in}^{k},{\bar{Ex}}_{ij}^{k}\right],En_{in}^{k},He_{in}^{k}\right)$$ (27)

Where $\tilde{C}\left(\left[y_{in}^{kL},y_{in}^{kU}\right]\right)$ is the interval cloud evaluation of $\left[y_{in}^{kL},y_{in}^{kU}\right]$.

**Step 3:** Compute the rough could evaluations:

While the cloud model can represent the uncertainty in expert judgments, it does not address the uncertainty associated with market information. Rough set theory is needed to handle this type of uncertainty. Thus, further processing involving rough set theory is required. Using Eqs (15) to (23), the rough cloud importance of each sustainable agile design management practice can be determined as follows:

$$R\tilde{C}\left(\left[f_{n}^{kL},f_{n}^{kU}\right]\right)=\left(\left[{\underline{Ex}}_{n}^{\prime k},{\bar{Ex}}_{n}^{k}\right],En_{n}^{\prime k},He_{n}^{\prime k}\right)$$ (28)

In this equation, $R\tilde{C}\left(\left[f_{n}^{kL},f_{n}^{kU}\right]\right)$ represents the rough cloud importance of the nth practice provided by the *kth* expert. Similarly, the cloud assessments for product development plans related to each practice can be computed in the following manner:

$$R\tilde{C}\left(\left[y_{in}^{kL},y_{in}^{kU}\right]\right)=\left(\left[{\underline{Ex}}_{in}^{\prime k},{\bar{Ex}}_{in}^{k}\right],En_{in}^{\prime k},He_{in}^{\prime k}\right)$$ (29)

in which, $R\tilde{C}\left(\left[y_{in}^{kL},y_{in}^{kU}\right]\right)$ is the rough cloud assessment for $y_{in}^{k}$.

**Step 4:** Collect the rough cloud evaluations from different experts.

By applying the arithmetic mean method to combine individual rough cloud assessments, the overall rough cloud significance of SAPD practices can be determined as follows:

$$\begin{array}{l} R\tilde{C}\left(f_{i}\right)=\left(\left[\underline{Ex_{n}},{\bar{Ex}}_{j}\right],En_{n},He_{n}\right)\\ =\left(\left[\frac{1}{l}\sum_{k=1}^{l}{\underline{Ex}}_{n}^{\prime k},\frac{1}{l}\sum_{k=1}^{l}{\bar{Ex}}_{n}^{\prime k}\right],\sqrt{\frac{1}{l}\sum_{k=1}^{l}{\left(En_{n}^{\prime k}\right)}^{2}},\sqrt{\frac{1}{l}\sum_{k=1}^{l}{\left(He_{n}^{\prime k}\right)}^{2}}\right) \end{array}$$ (30)

in which $R\tilde{C}\left(f_{i}\right)$ is the group rough cloud importance of the *nth* SAPD practice.

Subsequently, the group rough cloud evaluation for product development plans under each practice can be calculated using the following method:

$$\begin{array}{l} R\tilde{C}\left(y_{in}\right)=\left(\left[{\underline{Ex}}_{in},{\bar{Ex}}_{in}\right],En_{in},He_{in}\right)\\ =\left(\left[\frac{1}{l}\sum_{k=1}^{l}Ex_{in}^{\prime k},\frac{1}{l}\sum_{k=1}^{l}{\bar{Ex}}_{in}^{k}\right],\sqrt{\frac{1}{l}\sum_{k=1}^{l}{\left(En_{in}^{\prime k}\right)}^{2}},\sqrt{\frac{1}{l}\sum_{k=1}^{l}{\left(He_{in}^{\prime k}\right)}^{2}}\right) \end{array}$$ (31)

in which $R\tilde{C}\left(y_{in}\right)$ represents the group rough cloud evaluation for *y*_*in*_.

#### 3.2.3. Determination of the weights of SAPD practices

**Step 1:** Find out the subjective weights of SAPD practices.

The formula for the subjective weighting of SAPD practices is as follows:

$$W_{n}^{s}=Ex_{n}/\sum_{n=1}^{j}Ex_{n}$$ (32)

In the equation, $W_{n}^{s}$ represents the subjective weight of the *nth* practice, hence: $Ex_{n}=\left({\underline{Ex}}_{n}+{\bar{Ex}}_{n}\right)/2$.

**Step 2:** Find out the objective weights of SAPD practices.

The objective weights of SAPD practices are determined through the concept of statistical variance [63]. The steps involved in their calculation are outlined as follows:

$$R\tilde{C}{\left(y_{in}\right)}_{mean}=\frac{1}{m}\sum_{i=1}^{m}R\tilde{C}\left(y_{in}\right)=\left(\left[\frac{1}{m}\sum_{i=1}^{m}\underline{Ex_{in}},\frac{1}{m}\sum_{i=1}^{m}\bar{Ex_{in}}\right],\sqrt{\frac{1}{m}\sum_{i=1}^{m}{\left(En_{in}\right)}^{2}},\sqrt{\frac{1}{m}\sum_{i=1}^{m}{\left(He_{in}\right)}^{2}}\right)$$ (33)

$$V_{n}=\frac{1}{m}\sum_{j=1}^{m}\left(\left[\underline{Ex_{in}},\bar{Ex_{in}}\right],En_{in},He_{in}\right)$$ (34)

$$W_{n}^{o}=V_{n}/\sum_{n=1}^{j}V_{n}$$ (35)

where $R\tilde{C}{\left(y_{in}\right)}_{mean}$ is the mean value. *V*_*n*_ is the variance of agile sustainable design methods rough cloud evaluations under the *nth* method. $W_{n}^{o}$ is the objective weight of the *nth* method.

**Step 3:** Compute the combination weights of SAPD practices.

Then, the combination weight of the *nth* practice is calculated by:

$$W_{n}^{C}=\frac{W_{n}^{S}\times W_{n}^{O}}{\sum_{n=1}^{j}W_{n}^{S}\times W_{n}^{O}}$$ (36)

in which $W_{n}^{C}$ represents the combination weight.

#### 3.2.4. Product development method based on an extended TOPSIS

**Step 1:** Normalize the rough cloud decision matrix

Due to variations in the dimensions and magnitudes of the criteria, the cloud decision matrix should be normalized to make it comparable. However, in this paper, as the effective domain is already between 0 and 1, there is no need for further normalization.

**Step 2:** Compute the weighted rough cloud decision matrix.

The weighted rough cloud assessment $R\tilde{C}\left(y_{in}^{\prime }\right)$ is calculated by

$$R\tilde{C}\left(y_{in}^{\prime }\right)=w_{n}^{C}\times R\tilde{C}\left(y_{in}\right)=w_{n}^{C}\times \left(\left[\underline{Ex_{in}},\bar{Ex_{in}}\right],En_{in},He_{in}\right)=\left(\left[\underline{Ex_{in}^{\prime }},\bar{Ex_{in}^{\prime }}\right],En_{in}^{\prime },He_{in}^{\prime }\right)$$ (37)

**Step 3:** Recognize the positive and negative solutions.

Based on the weighted rough cloud decision matrix, the positive and negative solutions can be identified as follows:

$$R{\tilde{C}}^{+}\left(y_{in}^{\prime }\right)=\left(\left[max_{i}\underline{Ex_{in}^{\prime }},max_{i}\bar{Ex_{in}^{\prime }}\right],min_{i}En_{in}^{\prime },min_{i}He_{in}^{\prime }\right)$$ (38)

$$R{\tilde{C}}^{-}\left(y_{in}^{\prime }\right)=\left(\left[min_{i}\underline{Ex_{in}^{\prime }},min_{i}\bar{Ex_{in}^{\prime }}\right],max_{i}En_{in}^{\prime },max_{i}He_{in}^{\prime }\right)$$ (39)

in which, $R{\tilde{C}}^{+}\left(y_{in}^{\prime }\right)$ represents the positive ideal solution and $R{\tilde{C}}^{-}\left(y_{in}^{\prime }\right)$ is the negative ideal solution.

**Step 4.** Compute the distance between each market and ideal solution.

Using Eq (8), the distance between each market and the ideal solution can be calculated as:

$$d_{i}^{+}=\sqrt{\sum_{n=1}^{j}{\left[d\left(R\tilde{C}\left(y_{in}^{\prime }\right),R{\tilde{C}}^{+}\left(y_{n}^{\prime }\right)\right)\right]}^{2}}$$ (40)

$$d_{i}^{-}=\sqrt{\sum_{n=1}^{j}{\left[d\left(R\tilde{C}\left(y_{in}^{\prime }\right),R{\tilde{C}}^{+}\left(y_{n}^{\prime }\right)\right)\right]}^{2}}$$ (41)

in which $d_{i}^{+}$, and $d_{i}^{-}$ are the distance between the *ith* market and positive ideal solutions and negative ideal solution, respectively.

**Step 5:** obtain the closeness coefficient of each market.

Subsequently, the closeness coefficient for each market can be computed as follows:

$$CC_{i}=\frac{d_{i}^{-}}{d_{i}^{+}+d_{i}^{-}}$$ (42)

in which *CC*_*i*_ is the closeness coefficient of the *ith* market, which is the basis of markets’ ranking. The bigger the *CC*_*i*_, the better performance of the *ith* market (*i* = 1,2,…,*j*).

## 4. Result of case study

The study demonstrates the effectiveness of using a rough cloud methodology for the selection of an agile sustainable design method, as illustrated in a case study involving the development of a kitchen faucet.

### 4.1. Product development description

This study centers on a Chinese private technology company specializing in the development of showerheads, basin faucets, and kitchen faucets. The company’s primary mission is to advance water conservation by manufacturing showers with water-saving capabilities. Balancing plentiful water flow and a superior bathing experience is crucial for water-saving showers. While developing their products, the company encountered technical hurdles stemming from various design choices. Recognizing the necessity to expedite the development of their kitchen faucet, the company understood the importance of incorporating sustainable design principles and adopting suitable agile methodologies.

To achieve both product development agility and product sustainability, the company formed an expert panel tasked with evaluating the relative importance of sustainable design methods and agile management practices. The panel consists of 10 experts from within the organization, including 4 with more than a decade of academic and practical experience. In addition, one expert hails from the company’s sales department, another from the procurement department, and 4 from the project department. Furthermore, all panel members possess a minimum of five years of relevant work experience. These five experts will be responsible for selecting appropriate agile management approaches and sustainable design methods for this product development initiative based on their individual knowledge and expertise.

### 4.2. The application processes

Through a literature review, sustainable design methods and agile management methods have been compiled and are presented in Tables 1 and 2. In the following section, an application based on the steps of the rough cloud model as described in Section 3.2 will be demonstrated.

**Table 1:** Experts’ rough cloud preferences for the SAPD practices.

Practices	*k*_1_	*k*_2_	*k*_3_	⋯	*k*_10_
Sd_1_	([0.634, 0.974], 0.058, 0.027)	([0.520, 0.825], 0.042, 0.024)	([0.565, 0.826], 0.046, 0.26)	⋯	([0.345, 0.782], 0.042, 0.024)
Sd_2_	([0.683, 0.974], 0.058, 0.027)	([0.731,0.852], 0.047, 0.027)	([0.622, 0.814], 0.044, 0.025)	⋯	([0.521, 0.762], 0.038, 0.022)
Sd_3_	([0.578, 0.788],0.047, 0.026)	([0.620, 0.856], 0.044, 0.026)	([0.562, 0.786], 0.038, 0.022)	⋯	([0.552, 0.812], 0.044, 0.025)
Sd_4_	([0.512, 0.723], 0.033, 0.020)	([0.644, 0.788], 0.038, 0.022)	([0.711, 0.921], 0.046, 0.028)	⋯	([0.741, 0.986], 0.058, 0.033)
Sd_5_	([0.668, 0.822], 0.046, 0.025)	([0.885, 1.000], 0.064, 0.035)	([0.845, 0.985], 0.064, 0.036)	⋯	([0.687, 0.866], 0.046, 0.027)
Sd_6_	([0.542, 0.714], 0.033, 0.019)	([0.489, 0.685], 0.036, 0.021)	([0.625,0.863], 0.048, 0.025)	⋯	([0.618, 0.867], 0.047, 0.026)
Sd_7_	([0.632,0.709],0.033, 0.017)	([0.645, 0.701], 0.033, 0.018)	([0.521, 0.668], 0.028, 0.018)	⋯	([0.642, 0.736], 0.033, 0.021)
Sd_8_	([0.542, 0.856], 0.044, 0.023)	([0.485, 0.683], 0.035, 0.019)	([0.582, 0.824], 0.038, 0.024)	⋯	([0.485, 0.683], 0.035, 0.019)
Sd_9_	([0.608, 0.763], 0.033, 0.021)	([0.502, 0.688], 0.030, 0.018)	([0.502, 0.688], 0.030, 0.018)	⋯	([0.542, 0.749], 0.033, 0.018)
Sd_10_	([0.687, 0.873], 0.044, 0.023)	([0.502, 0.767], 0.041, 0.022)	([0.718, 0.882], 0.053, 0.031)	⋯	([0.721, 0.842], 0.049, 0.030)
Sd_11_	([0.574, 0.768], 0.041, 0.024)	([0.612, 0.763], 0.048, 0.026)	([0.568, 0.762], 0.038, 0.022)	⋯	([0.564, 0.781], 0.038, 0.024)
Sd_12_	([0.660, 1.000], 0.058, 0.028)	([0.541, 0.859], 0.043, 0.025)	([0.588, 0.860], 0.048, 0.027)	⋯	([0.359, 0.814], 0.044, 0.025)
Sd_13_	([0.711, 1.000], 0.060, 0.031)	([0.761, 0.887], 0.048, 0.028)	([0.647, 0.847], 0.046, 0.022)	⋯	([0.542, 0.793], 0.040, 0.023)
Sd_14_	([0.601, 0.821], 0.048, 0.027)	([0.645, 0.891], 0.045, 0.027)	([0.585, 0.818], 0.039, 0.022)	⋯	([0.574, 0.845], 0.046, 0.026)
Sd_15_	([0.533, 0.752], 0.034, 0.021)	([0.671, 0.821], 0.039, 0.022)	([0.740, 0.958], 0.048, 0.029)	⋯	([0.772, 1.000], 0.058, 0.034)
Sd_16_	([0.695, 0.856], 0.047, 0.026)	([0.821, 0.996], 0.061, 0.036)	([0.881, 1.000], 0.064, 0.037)	⋯	([0.715, 0.902], 0.048, 0.028)
Sd_17_	([0.564, 0.743], 0.034, 0.019)	([0.511, 0.713], 0.037, 0.022)	([0.651, 0.898], 0.050, 0.026)	⋯	([0.643, 0.902], 0.048, 0.027)
Sd_18_	([0.658, 0.738], 0.034, 0.017)	([0.671, 0.731], 0.034, 0.018)	([0.542, 0.695], 0.029, 0.018)	⋯	([0.668, 0.766], 0.034, 0.022)
Sd_19_	([0.564, 0.891], 0.045, 0.024)	([0.505, 0.711], 0.036, 0.019)	([0.606, 0.857], 0.039, 0.024)	⋯	([0.510, 0.711], 0.036, 0.019)
Sd_20_	([0.633, 0.794], 0.034, 0.022)	([0.522,0.716], 0.031, 0.018)	([0.522, 0.716], 0.031, 0.018)	⋯	([0.564, 0.779], 0.034, 0.018)
Sd_21_	([0.715, 0.910], 0.045, 0.024)	([0.522, 0.798], 0.042, 0.023)	([0.747, 0.918], 0.055, 0.032)	⋯	([0.751, 0.876], 0.051, 0.031)
Sd_22_	([0.597, 0.801], 0.042, 0.025)	([0.637, 0.794], 0.049, 0.027)	([0.591, 0.793], 0.039, 0.023)	⋯	([0.587, 0.813], 0.040, 0.025)
Sd_23_	([0.647, 0.994], 0.058, 0.028)	([0.531, 0.842], 0.042, 0.024)	([0.576, 0.843], 0.046, 0.026)	⋯	([0.352, 0.798], 0.043, 0.024)
Sd_24_	([0.697, 0.994), 0.059, 0.027)	([0.746, 0.869], 0.047, 0.027)	([0.634, 0.831], 0.044, 0.025)	⋯	([0.532, 0.778], 0.038, 0.022)
Am_1_	([0.589, 0.804], 0.047, 0.026)	([0.632, 0.873], 0.044, 0.026)	([0.573, 0.802], 0.038, 0.022)	⋯	([0.563, 0.828], 0.045, 0.025)
Am_2_	([0.522, 0.737], 0.033, 0.020)	([0.657, 0.804], 0.038, 0.022)	([0.725, 0.939], 0.046, 0.028)	⋯	([0.756, 1.000], 0.058, 0.033)
Am_3_	([0.682, 0.839], 0.046, 0.025)	([0.903, 1.000], 0.061, 0.035)	([0.862, 1.000], 0.062, 0.036)	⋯	([0.701, 0.883], 0.047, 0.027)
Am_4_	([0.553, 0.728], 0.033, 0.019)	([0.501, 0.699], 0.036, 0.022)	([0.637, 0.881], 0.048, 0.025)	⋯	([0.631, 0.884], 0.047, 0.026)
Am_5_	([0.645, 0.723], 0.033, 0.017)	([0.658, 0.715], 0.033, 0.018)	([0.531, 0.682], 0.028, 0.018)	⋯	([0.655, 0.751], 0.033, 0.021)
Am_6_	([0.553, 0.873], 0.044, 0.023)	([0.495, 0.697], 0.035, 0.019)	([0.594, 0.841], 0.038, 0.024)	⋯	([0.495, 0.697], 0.035, 0.019)
Am_7_	([0.621, 0.778], 0.033, 0.021)	([0.512, 0.702], 0.031, 0.018)	([0.512, 0.702], 0.031, 0.018)	⋯	([0.553, 0.764], 0.034, 0.018)
Am_8_	([0.701, 0.891], 0.044, 0.023)	([0.512, 0.783], 0.042, 0.022)	([0.732, 0.901], 0.054, 0.032)	⋯	([0.736, 0.859], 0.050, 0.036)

**Table 2:** Experts’ rough cloud ratings of design plan A1 with respect to SAPD practices.

Practices	*k*_1_	*k*_2_	*k*_3_	⋯	*k*_10_
Sd_1_	([0.626, 0.962], 0.057, 0.027)	([0.514, 0.815], 0.041, 0.024)	([0.558, 0.816], 0.045, 0.026)	⋯	([0.341, 0.773], 0.041, 0.024)
Sd_2_	([0.675, 0.962], 0.057, 0.027)	([0.722, 0.842], 0.046, 0.027)	([0.614, 0.804], 0.043, 0.025)	⋯	([0.515, 0.753], 0.038, 0.022)
Sd_3_	([0.571, 0.778], 0.046, 0.026)	([0.612, 0.846], 0.043, 0.026)	([0.555, 0.776], 0.037, 0.022)	⋯	([0.545, 0.802], 0.043, 0.025)
Sd_4_	([0.506, 0.714], 0.033, 0.020)	([0.479, 0.675], 0.034, 0.019)	([0.702, 0.910], 0.045, 0.027)	⋯	([0.732, 0.974], 0.057, 0.032)
Sd_5_	([0.660, 0.812], 0.045, 0.025)	([0.874, 0.988], 0.063, 0.035)	([0.835, 0.973], 0.063, 0.035)	⋯	([0.679, 0.855], 0.045, 0.033)
Sd_6_	([0.535, 0.705], 0.033, 0.019)	([0.483, 0.677], 0.036, 0.021)	([0.617, 0.852], 0.047, 0.025)	⋯	([0.610, 0.856], 0.046, 0.026)
Sd_7_	([0.624, 0.701], 0.033, 0.017)	([0.637, 0.692], 0.033, 0.018)	([0.514, 0.607], 0.027, 0.018)	⋯	([0.634, 0.727], 0.032, 0.021)
Sd_8_	([0.535, 0.846], 0.043, 0.023)	([0.479, 0.675], 0.034, 0.019)	([0.575, 0.813], 0.038, 0.024)	⋯	([0.479, 0.675], 0.034, 0.021)
Sd_9_	([0.601, 0.754], 0.033, 0.021)	([0.496, 0.680], 0.030, 0.018)	([0.495, 0.679], 0.029, 0.018)	⋯	([0.535, 0.740], 0.032, 0.018)
Sd_10_	([0.679, 0.862], 0.043, 0.023)	([0.496, 0.758], 0.040, 0.022)	([0.709, 0.871], 0.052, 0.031)	⋯	([0.712, 0.831], 0.048, 0.030)
Sd_11_	([0.567, 0.759], 0.041, 0.024)	([0.604, 0.754], 0.047, 0.026)	([0.561, 0.753], 0.038, 0.022)	⋯	([0.557, 0.772], 0.038, 0.024)
Sd_12_	([0.652, 1.000], 0.059, 0.028)	([0.535, 0.849], 0.043, 0.025)	([0.581, 0.849], 0.047, 0.027)	⋯	([0.355, 0.804], 0.043, 0.025)
Sd_13_	([0.703, 1.000], 0.060, 0.028)	([0.752, 0.876], 0.048, 0.028)	([0.640, 0.837], 0.045, 0.026)	⋯	([0.535, 0.783], 0.039, 0.023)
Sd_14_	([0.595, 0.811], 0.048, 0.027)	([0.638, 0.880], 0.045, 0.027)	([0.578, 0.808], 0.039, 0.023)	⋯	([0.568, 0.835], 0.045, 0.026)
Sd_15_	([0.527, 0.744], 0.034, 0.021)	([0.662, 0.811], 0.039, 0.023)	([0.731, 0.947], 0.047, 0.029)	⋯	([0.762, 1.000], 0.059, 0.034)
Sd_16_	([0.687, 0.846], 0.047, 0.026)	([0.910, 1.000], 0.066, 0.036)	([0.869, 1.000], 0.066, 0.037)	⋯	([0.707, 0.891], 0.047, 0.028)
Sd_17_	([0.558, 0.734], 0.034, 0.020)	([0.503, 0.704], 0.037, 0.022)	([0.643, 0.887], 0.049, 0.026)	⋯	([0.636, 0.892], 0.048, 0.027)
Sd_18_	([0.650, 0.729], 0.034, 0.017)	([0.663, 0.721], 0.034, 0.019)	([0.536, 0.687], 0.029, 0.018)	⋯	([0.660, 0.757], 0.034, 0.022)
Sd_19_	([0.558, 0.880], 0.045, 0.024)	([0.499, 0.703], 0.036, 0.020)	([0.599, 0.848], 0.039, 0.025)	⋯	([0.499, 0.703], 0.036, 0.019)
Sd_20_	([0.625, 0.785], 0.034, 0.022)	([0.516, 0.708], 0.031, 0.019)	([0.516, 0.708], 0.031, 0.018)	⋯	([0.557, 0.770], 0.034, 0.018)
Sd_21_	([0.707, 0.898], 0.045, 0.024)	([0.516, 0.789], 0.042, 0.023)	([0.739, 0.907], 0.054, 0.032)	⋯	([0.742, 0.866], 0.050, 0.031)
Sd_22_	([0.590, 0.790], 0.042, 0.025)	([0.629, 0.785], 0.049, 0.027)	([0.584, 0.784], 0.039, 0.023)	⋯	([0.580, 0.803], 0.039, 0.025)
Sd_23_	([0.639, 0.982], 0.058, 0.027)	([0.524, 0.832], 0.042, 0.024)	([0.569, 0.832], 0.046, 0.026)	⋯	([0.348, 0.788], 0.042, 0.024)
Sd_24_	([0.689, 0.982], 0.058, 0.027)	([0.737, 0.859], 0.047, 0.027)	([0.627, 0.820], 0.044, 0.025)	⋯	([0.525, 0.786], 0.038, 0.022)
Am_1_	([0.582, 0.794], 0.047, 0.026)	([0.625, 0.863], 0.044, 0.026)	([0.567, 0.792], 0.038, 0.022)	⋯	([0.556, 0.819], 0.044, 0.025)
Am_2_	([0.516, 0.729], 0.033, 0.020)	([0.649, 0.794], 0.038, 0.021)	([0.717, 0.928], 0.046, 0.028)	⋯	([0.747, 0.994], 0.058, 0.033)
Am_3_	([0.674, 0.829], 0.046, 0.025)	([0.892, 1.000], 0.064, 0.035)	([0.852, 0.993], 0.064, 0.036)	⋯	([0.693, 0.873], 0.046, 0.027)
Am_4_	([0.546, 0.720], 0.033, 0.019)	([0.493, 0.691], 0.036, 0.021)	([0.630, 0.870], 0.048, 0.025)	⋯	([0.623, 0.874], 0.047, 0.026)
Am_5_	([0.637, 0.715], 0.033, 0.017)	([0.650, 0.707], 0.033, 0.018)	([0.525, 0.673], 0.028, 0.018)	⋯	([0.647, 0.742], 0.033, 0.021)
Am_6_	([0.546, 0.863], 0.044, 0.023)	([0.489, 0.689], 0.035, 0.019)	([0.587, 0.831], 0.038, 0.024)		([0.489, 0.689], 0.035, 0.019)
Am_7_	([0.613, 0.769], 0.033, 0.021)	([0.506, 0.694], 0.030, 0.018)	([0.506, 0.693], 0.030, 0.018)		([0.546, 0.755], 0.033, 0.018)
Am_8_	([0.692, 0.880], 0.044, 0.023)	([0.506, 0.773], 0.041, 0.022)	([0.723, 0.889], 0.053, 0.031)		([0.727, 0.849], 0.049, 0.030)

#### 4.2.1. Establish design plans and evaluation metrics

During the initial phases of product development, enterprise designers suggested three design concepts: (1) Implement induction technology to decrease water output when individuals are not directly under the rain head; (2) Incorporate suction technology to enhance the infusion of air into the water; (3) Conduct innovative structural research and development, disperse water droplets through internal structures, utilize minimal water outlet holes, and establish an ample bathing atmosphere with reduced water usage.

#### 4.2.2. Assessment of SAPD practices and methods

**Step 1:** Obtain the interval linguistic evaluations for practices and methods.

The SAPD practices employed for the purpose of selecting a sustainable agile method, as illustrated in Tables A.1-A.2 in S1 File, have been acknowledged through a review of existing literature. Furthermore, experts have confirmed their appropriateness. Subsequently, the company devised a questionnaire and distributed it to ten experts. The tables in question, namely Tables A.6-A.7 in S1 File (present interval linguistic assessments for both practices and methods, considering the inherent uncertainty in the judgments of the experts. Following the utilization of Eqs (26) and (27), along with the definitions provided in Sections 5 and 6, the qualitative evaluations are transformed into relative quantitative assessments, specifically in the form of interval clouds.

Step 2: Calculate the rough cloud assessments.

Throughout the judgment process, there was also a presence of interpersonal uncertainty. In this phase, rough set theory is applied to investigate this type of uncertainty. Utilizing Eqs (17)–(23) and (28)-(29), rough cloud assessments are computed, taking into consideration both intrapersonal and interpersonal uncertainty. Table 1 displays the rough cloud preferences for the SAPD practices. Due to space constraints, Table 2 exclusively presents the rough cloud rating for design plan A1.

Step 3. Aggregate the rough cloud evaluations from different experts.

By applying Eqs (30) and (31), the rough cloud assessments offered by various experts are consolidated to form group rough cloud evaluations. Tables 3 and 4 present the group rough cloud preferences for practices and the group rough cloud ratings for design plans, which essentially constitute the group rough cloud decision matrix.

**Table 3:** Group rough cloud preferences of SAPD practices.

Practices	Group preferences
Sd_1_	([0.592, 0.909], 0.054, 0.025)
Sd_2_	([0.638, 0.909], 0.054, 0.025)
Sd_3_	([0.540, 0.736], 0.044, 0.024)
Sd_4_	([0.478, 0.675], 0.031, 0.018)
Sd_5_	([0.624, 0.768], 0.043, 0.023)
Sd_6_	([0.506, 0.667], 0.031, 0.018)
Sd_7_	([0.590, 0.662], 0.031, 0.016)
Sd_8_	([0.506, 0.799], 0.041, 0.021)
Sd_9_	([0.568, 0.713], 0.031, 0.020)
Sd_10_	([0.642, 0.815], 0.041, 0.021)
Sd_11_	([0.536, 0.717], 0.038, 0.022)
Sd_12_	([0.617, 0.947], 0.056, 0.026)
Sd_13_	([0.664, 0.747], 0.056, 0.026)
Sd_14_	([0.562, 0.766], 0.045, 0.025)
Sd_15_	([0.498, 0.703], 0.032, 0.019)
Sd_16_	([0.649, 0.799], 0.044, 0.024)
Sd_17_	([0.527, 0.694], 0.032, 0.018)
Sd_18_	([0.614, 0.689], 0.032, 0.016)
Sd_19_	([0.527, 0.832], 0.043, 0.022)
Sd_20_	([0.591, 0.742], 0.032, 0.020)
Sd_21_	([0.668, 0.849], 0.043, 0.022)
Sd_22_	([0.558, 0.747], 0.040, 0.023)
Sd_23_	([0.604, 0.928], 0.055, 0.026)
Sd_24_	([0.651, 0.928], 0.055, 0.026)
Am_1_	([0.551, 0.751], 0.045, 0.025)
Am_2_	([0.488, 0.689], 0.031, 0.019)
Am_3_	([0.647, 0.784], 0.044, 0.024)
Am_4_	([0.517, 0.681], 0.031, 0.018)
Am_5_	([0.602, 0.676], 0.031, 0.016)
Am_6_	([0.517, 0.816], 0.042, 0.022)
Am_7_	([0.579, 0.727], 0.031, 0.020)
Am_8_	([0.655, 0.832], 0.042, 0.022)

**Table 4:** Group rough cloud ratings of design plans with respect to SAPD practices.

Practices	*A*1	*A*2	*A*3
Sd_1_	([0.605, 0.929], 0.055, 0.025)	([0.496, 0.787], 0.040, 0.023)	([0.539, 0.788], 0.044, 0.025)
Sd_2_	([0.652, 0.929], 0.055, 0.026)	([0.697, 0.813], 0.045, 0.026)	([0.593, 0.777], 0.042, 0.024)
Sd_3_	([0.551, 0.752], 0.045, 0.025)	([0.592, 0.816], 0.042, 0.025)	([0.536, 0.750], 0.036, 0.021)
Sd_4_	([0.489, 0.690], 0.031, 0.019)	([0.614, 0.752], 0.036, 0.021)	([0.678, 0.879], 0.044, 0.027)
Sd_5_	([0.637, 0.784], 0.044, 0.024)	([0.844, 0.549], 0.061, 0.033)	([0.806, 0.940], 0.061, 0.034)
Sd_6_	([0.517, 0.681], 0.031, 0.018)	([0.467, 0.654], 0.034, 0.020)	([0.596, 0.823], 0.046, 0.024)
Sd_7_	([0.603, 0.676], 0.031, 0.016)	([0.615, 0.669], 0.031, 0.017)	([0.497, 0.637], 0.027, 0.017)
Sd_8_	([0.517, 0.817], 0.042, 0.022)	([0.463, 0.652], 0.033, 0.018)	([0.555, 0.786], 0.036, 0.023)
Sd_9_	([0.580, 0.727], 0.031, 0.020)	([0.479, 0.656], 0.029, 0.017)	([0.479, 0.656], 0.029, 0.017)
Sd_10_	([0.655, 0.833], 0.042, 0.022)	([0.479, 0.732], 0.039, 0.021)	([0.685, 0.842], 0.051, 0.030)
Sd_11_	([0.548, 0.733], 0.039, 0.023)	([0.584, 0.728], 0.046, 0.025)	([0.542, 0.727], 0.036, 0.021)
Sd_12_	([0.629, 0.967], 0.058, 0.027)	([0.517, 0.819], 0.042, 0.024)	([0.561, 0.821], 0.046, 0.026)
Sd_13_	([0.679, 0.968], 0.058, 0.027)	([0.726, 0.846], 0.047, 0.027)	([0.618, 0.809], 0.044, 0.025)
Sd_14_	([0.574, 0.783], 0.047, 0.026)	([0.616, 0.850], 0.044, 0.026)	([0.558, 0.781], 0.038, 0.022)
Sd_15_	([0.509, 0.718], 0.032, 0.020)	([0.640, 0.783], 0.038, 0.022)	([0.706, 0.915], 0.046, 0.028)
Sd_16_	([0.664, 0.817], 0.046, 0.025)	([0.879, 0.993], 0.064, 0.035)	([0.839, 0.979], 0.064, 0.036)
Sd_17_	([0.538, 0.709], 0.033, 0.019)	([0.486, 0.681], 0.036, 0.021)	([0.621, 0.857], 0.048, 0.025)
Sd_18_	([0.628, 0.704], 0.033, 0.017)	([0.641, 0.696], 0.033, 0.018)	([0.518, 0.664], 0.028, 0.018)
Sd_19_	([0.538, 0.850], 0.044, 0.023)	([0.482, 0.679], 0.035, 0.019)	([0.578, 0.819], 0.038, 0.024)
Sd_20_	([0.604, 0.758], 0.033, 0.021)	([0.499, 0.684], 0.030, 0.018)	([0.499, 0.684], 0.030, 0.018)
Sd_21_	([0.683, 0.867], 0.044, 0.023)	([0.499, 0.762], 0.030, 0.018)	([0.713, 0.876], 0.053, 0.031)
Sd_22_	([0.570, 0.763], 0.041, 0.024)	([0.608, 0.758], 0.048, 0.026)	([0.564, 0.757], 0.038, 0.022)
Sd_23_	([0.617, 0.948], 0.056, 0.026)	([0.506, 0.803], 0.041, 0.023)	([0.550, 0.804], 0.045, 0.025)
Sd_24_	([0.665, 0.948], 0.056, 0.026)	([0.712, 0.830], 0.046, 0.026)	([0.606, 0.793], 0.043, 0.024)
Am_1_	([0.563, 0.767], 0.046, 0.025)	([0.604, 0.834], 0.043, 0.025)	([0.547, 0.765], 0.037, 0.021)
Am_2_	([0.499, 0.704], 0.032, 0.019)	([0.627, 0.767], 0.037, 0.021)	([0.692, 0.897], 0.045, 0.027)
Am_3_	([0.650, 0.800], 0.045, 0.024)	([0.862, 0.974], 0.062, 0.034)	([0.883, 0.959], 0.062, 0.035)
Am_4_	([0.528, 0.695], 0.032, 0.019)	([0.476, 0.667], 0.035, 0.020)	([0.609, 0.840], 0.047, 0.024)
Am_5_	([0.615, 0.690], 0.032, 0.017)	([0.628, 0.683], 0.032, 0.017)	([0.507, 0.650], 0.027, 0.018)
Am_6_	([0.528, 0.834], 0.043, 0.022)	([0.472, 0.665], 0.034, 0.019)	([0.567, 0.802], 0.037, 0.023)
Am_7_	([0.592, 0.743], 0.032, 0.020)	([0.489, 0.670], 0.029, 0.018)	([0.489, 0.670], 0.029, 0.018)
Am_8_	([0.669, 0.850], 0.043, 0.022)	([0.489, 0.747], 0.040, 0.021)	([0.699, 0.858], 0.052, 0.030)

#### 4.2.3. Weight determination of SAPD practices

To start, the subjective weights of SAPD practices are determined in accordance with Eqs (32). Subsequently, by applying Eqs (33)–(35), the objective weights of these practices are derived. Finally, the combination weights are computed using Eq (36). The outcomes of the weight calculations are displayed in Table 5.

**Table 5:** Weight of SAPD practices.

Practices	Subjective weights	Objective weights	Combination weights
Sd_1_	0.076	0.093	0.101
Sd_2_	0.082	0.070	0.78
Sd_3_	0.074	0.060	0.064
Sd_4_	0.068	0.107	0.098
Sd_5_	0.088	0.114	0.121
Sd_6_	0.092	0.064	0.073
Sd_7_	0.085	0.041	0.045
Sd_8_	0.086	0.043	0.052
Sd_9_	0.071	0.061	0.061
Sd_10_	0.076	0.023	0.024
Sd_11_	0.062	0.068	0.056
Sd_12_	0.046	0.036	0.025
Sd_13_	0.052	0.083	0.054
Sd_14_	0.058	0.054	0.046
Sd_15_	0.079	0.096	0.105
Sd_16_	0.085	0.072	0.812
Sd_17_	0.077	0.062	0.066
Sd_18_	0.071	0.111	0.102
Sd_19_	0.091	0.118	0.125
Sd_20_	0.095	0.066	0.076
Sd_21_	0.088	0.042	0.046
Sd_22_	0.089	0.044	0.054
Sd_23_	0.073	0.063	0.063
Sd_24_	0.079	0.023	0.024
Am_1_	0.064	0.071	0.058
Am_2_	0.047	0.037	0.026
Am_3_	0.054	0.086	0.056
Am_4_	0.061	0.056	0.047
Am_5_	0.077	0.094	0.103
Am_6_	0.083	0.071	0.796
Am_7_	0.075	0.061	0.065
Am_8_	0.069	0.109	0.1000

#### 4.2.4. Sustainable design methods ranking based on an extended TOPSIS method. Step 1: Normalize the rough cloud decision matrix

Given that the domain ranges from 0 to 1, it is unnecessary to normalize the rough cloud decision matrix.

**Step 2:** Calculate the weighted rough cloud decision matrix.

Following Eq (37), the weighted rough cloud decision matrix is computed, and the results are presented in Table 6.

**Table 6:** Weighted Group rough cloud evaluations of design methods.

Practices	*A*1	*A*2	*A*3
Sd_1_	([0.046, 0.072], 0.008, 0.004)	([0.038, 0.060], 0.006, 0.003)	([0.042, 0.061], 0.007, 0.004)
Sd_2_	([0.051, 0.072], 0.008, 0.004)	([0.053, 0.062], 0.006, 0.004)	([0.046, 0.059], 0.007, 0.004)
Sd_3_	([0.042, 0.058], 0.007, 0.004)	([0.045, 0.063], 0.006, 0.004)	([0.041, 0.058], 0.006, 0.003)
Sd_4_	([0.038, 0.053], 0.004, 0.003)	([0.045, 0.063], 0.006, 0.004)	([0.052, 0.067], 0.007, 0.004)
Sd_5_	([0.049, 0.061], 0.006, 0.004)	([0.065, 0.074], 0.010, 0.005)	([0.062, 0.072], 0.009, 0.005)
Sd_6_	([0.041, 0.053], 0.005, 0.003)	([0.036, 0.054], 0.005, 0.003)	([0.046, 0.063], 0.007, 0.004)
Sd_7_	([0.046, 0.052], 0.005, 0.002)	([0.047, 0.051], 0.005, 0.002)	([0.038, 0.049], 0.004, 0.002)
Sd_8_	([0.039, 0.063], 0.006, 0.003)	([0.035, 0.050], 0.005, 0.003)	([0.042, 0.061], 0.005, 0.003)
Sd_9_	([0.044, 0.056], 0.005, 0.003)	([0.037, 0.051], 0.004, 0.003)	([0.036, 0.051], 0.004, 0.002)
Sd_10_	([0.051, 0.064], 0.006, 0.003)	([0.036, 0.056], 0.006, 0.003)	([0.053, 0.064], 0.008, 0.005)
Sd_11_	([0.042, 0.056], 0.007, 0.004]	([0.045, 0.056], 0.007, 0.003)	([0.042, 0.056], 0.005, 0.003)
Sd_12_	([0.048, 0.075], 0.010, 0.004)	([0.039, 0.063], 0.006, 0.004)	([0.043, 0.063], 0.007, 0.004)
Sd_13_	([0.052, 0.074], 0.091, 0.005)	([0.056, 0.065], 0.007, 0.004)	([0.047, 0.062], 0.007, 0.004)
Sd_14_	([0.044, 0.060], 0.007, 0.004)	([0.047, 0.065], 0.007, 0.004)	([0.043, 0.060], 0.006, 0.003)
Sd_15_	([0.039, 0.055], 0.005, 0.003)	([0.049, 0.060], 0.006, 0.003)	([0.054, 0.071], 0.007, 0.004)
Sd_16_	([0.051, 0.063], 0.007, 0.004)	([0.067, 0.076], 0.012, 0.005)	([0.065, 0.075], 0.098, 0.005)
Sd_17_	([0.042, 0.054], 0.005, 0.003)	([0.037, 0.052], 0.005, 0.003)	([0.047, 0.066], 0.007, 0.004)
Sd_18_	([0.048, 0.054], 0.005, 0.002)	([0.049, 0.054], 0.005, 0.003)	([0.039, 0.051], 0.004, 0.003)
Sd_19_	([0.042, 0.065], 0.007, 0.003)	([0.037, 0.052], 0.005, 0.003)	([0.044, 0.063], 0.006, 0.004)
Sd_20_	([0.046, 0.058], 0.005, 0.003)	([0.038, 0.052], 0.004, 0.003)	([0.038, 0.052], 0.005, 0.003)
Sd_21_	([0.052, 0.067], 0.007, 0.004)	([0.038, 0.058], 0.006, 0.003)	([0.055, 0.067], 0.008, 0.005)
Sd_22_	([0.044, 0.058], 0.006, 0.004)	([0.046, 0.058], 0.007, 0.004)	([0.043, 0.058], 0.006, 0.003)
Sd_23_	([0.047, 0.073], 0.091, 0.004)	([0.039, 0.062], 0.006, 0.004)	([0.042, 0.062], 0.007, 0.004)
Sd_24_	([0.051, 0.073], 0.008, 0.005)	([0.054, 0.064], 0.007, 0.004)	([0.047, 0.061], 0.007, 0.004)
Am_1_	([0.043, 0.059], 0.007, 0.004)	([0.046, 0.064], 0.006, 0.004)	([0.042, 0.059], 0.006, 0.003)
Am_2_	([0.038, 0.054], 0.005, 0.003)	([0.048, 0.059], 0.006, 0.003)	([0.053, 0.069], 0.007, 0.004)
Am_3_	([0.050, 0.061], 0.007, 0.003)	([0.066, 0.075], 0.010, 0.005)	([0.063, 0.074], 0.010, 0.006)
Am_4_	([0.041, 0.053], 0.005, 0.002)	([0.036, 0.051], 0.005, 0.003)	([0.046, 0.064], 0.007, 0.004)
Am_5_	([0.047, 0.053], 0.005, 0.003)	([0.048, 0.052], 0.005, 0.003)	([0.039, 0.050], 0.004, 0.003)
Am_6_	([0.041, 0.064], 0.006, 0.003)	([0.036, 0.051], 0.005, 0.003)	([0.043, 0.062], 0.006, 0.004)
Am_7_	([0.045, 0.057], 0.005, 0.003)	([0.037, 0.051], 0.004, 0.002)	([0.037, 0.052], 0.005, 0.003)
Am_8_	([0.052, 0.065], 0.007, 0.003)	([0.037, 0.057], 0.006, 0.003)	([0.054, 0.066], 0.008, 0.005)

**Step 3:** recognize the positive and negative ideal solution.

The positive and negative ideal solutions, as outlined in Table 7, are determined based on the weighted rough cloud decision matrix using Eqs (38) and (39).

**Table 7:** The positive and negative ideal solution.

Practices	Positive ideal solution	Negative ideal solution
Sd_1_	([0.054, 0.083], 0.010, 0.005)	([0.044, 0.071], 0.007, 0.004)
Sd_2_	([0.058, 0.084], 0.010, 0.005)	([0.062, 0.073], 0.008, 0.005)
Sd_3_	([0.049, 0.068], 0.008, 0.004)	([0.053, 0.073], 0.007, 0.004)
Sd_4_	([0.044, 0.062], 0.006, 0.003)	([0.055, 0.067], 0.006, 0.003)
Sd_5_	([0.057, 0.071], 0.008, 0.004)	([0.076, 0.086], 0.011, 0.006)
Sd_6_	([0.046, 0.061], 0.006, 0.003)	([0.042, 0.058], 0.006, 0.004)
Sd_7_	([0.054, 0.061], 0.006, 0.003)	([0.055, 0.060], 0.006, 0.003)
Sd_8_	([0.046, 0.073], 0.007, 0.004)	([0.042, 0.058], 0.006, 0.003)
Sd_9_	([0.052, 0.065], 0.006, 0.004)	([0.043, 0.059], 0.005, 0.003)
Sd_10_	([0.059, 0.075], 0.007, 0.004)	([0.043, 0.066], 0.007, 0.004)
Sd_11_	([0.049, 0.066], 0.007, 0.004)	([0.052, 0.065], 0.008, 0.004)
Sd_12_	([0.056, 0.087], 0.010, 0.005)	([0.046, 0.073], 0.007, 0.004)
Sd_13_	([0.061, 0.087], 0.010, 0.005)	([0.065, 0.076], 0.008, 0.005)
Sd_14_	([0.052, 0.071], 0.008, 0.005)	([0.057, 0.071], 0.008, 0.005)
Sd_15_	([0.046, 0.064], 0.006, 0.004)	([0.057, 0.061], 0.007, 0.004)
Sd_16_	([0.059, 0.074], 0.008, 0.004)	([0.079, 0.089], 0.011, 0.006)
Sd_17_	([0.048, 0.063], 0.006, 0.003)	([0.043, 0.061], 0.006, 0.004)
Sd_18_	([0.056, 0.063], 0.006, 0.003)	([0.057, 0.062], 0.006, 0.003)
Sd_19_	([0.048, 0.076], 0.008, 0.004)	([0.043, 0.061], 0.006, 0.003)
Sd_20_	([0.054, 0.068], 0.006, 0.004)	([0.045, 0.062], 0.005, 0.003)
Sd_21_	([0.062, 0.078], 0.008, 0.004)	([0.044, 0.068], 0.007, 0.004)
Sd_22_	([0.051, 0.068], 0.007, 0.004)	([0.054, 0.068], 0.091, 0.005)
Sd_23_	([0.055, 0.085], 0.010, 0.005)	([0.045, 0.072], 0.007, 0.004)
Sd_24_	([0.060, 0.085], 0.010, 0.005)	([0.064, 0.075], 0.008, 0.005)
Am_1_	([0.051, 0.069], 0.008, 0.004)	([0.054, 0.075], 0.008, 0.005)
Am_2_	([0.045, 0.063], 0.006, 0.003)	([0.056, 0.069], 0.006, 0.004)
Am_3_	([0.058, 0.072], 0.008, 0.004)	([0.077, 0.087], 0.011, 0.006)
Am_4_	([0.047, 0.062], 0.006, 0.003)	([0.042, 0.060], 0.006, 0.004)
Am_5_	([0.055, 0.062], 0.006, 0.003)	([0.056, 0.061], 0.006, 0.003)
Am_6_	([0.047, 0.075], 0.008, 0.004)	([0.043, 0.060], 0.006, 0.003)
Am_7_	([0.053, 0.067], 0.006, 0.004)	([0.044, 0.060], 0.005, 0.003)
Am_8_	([0.060, 0.076], 0.008, 0.004)	([0.044, 0.067], 0.007, 0.004)

**Step 4:** Calculate the distance between each design and the ideal solution.

Subsequently, the distances between the design methods and the positive and negative ideal solutions are calculated using Eqs (40) and (41), resulting in the values presented in Table 8.

**Table 8:** The distance.

Design plans	Distance from the positive ideal solution	Distance from the negative ideal solution
*A*1	0.042	0.058
*A*2	0.054	0.046
*A*3	0.063	0.037

**Step 5:** obtain the closeness coefficient of each design plan.

Ultimately, the closeness coefficient of each design plan is obtained using Eq (42), which serves as the foundation for ranking the sustainability of the design plans. The resulting values are displayed in Table 9.

**Table 9:** Closeness coefficients and design plans’ sustainability ranks.

Design plans	Closeness coefficient	Rank
*A*1	0.648	2
*A*2	0.562	3
*A*3	0.678	1

#### 4.2.5. Comparative analysis

**Fig 2:**
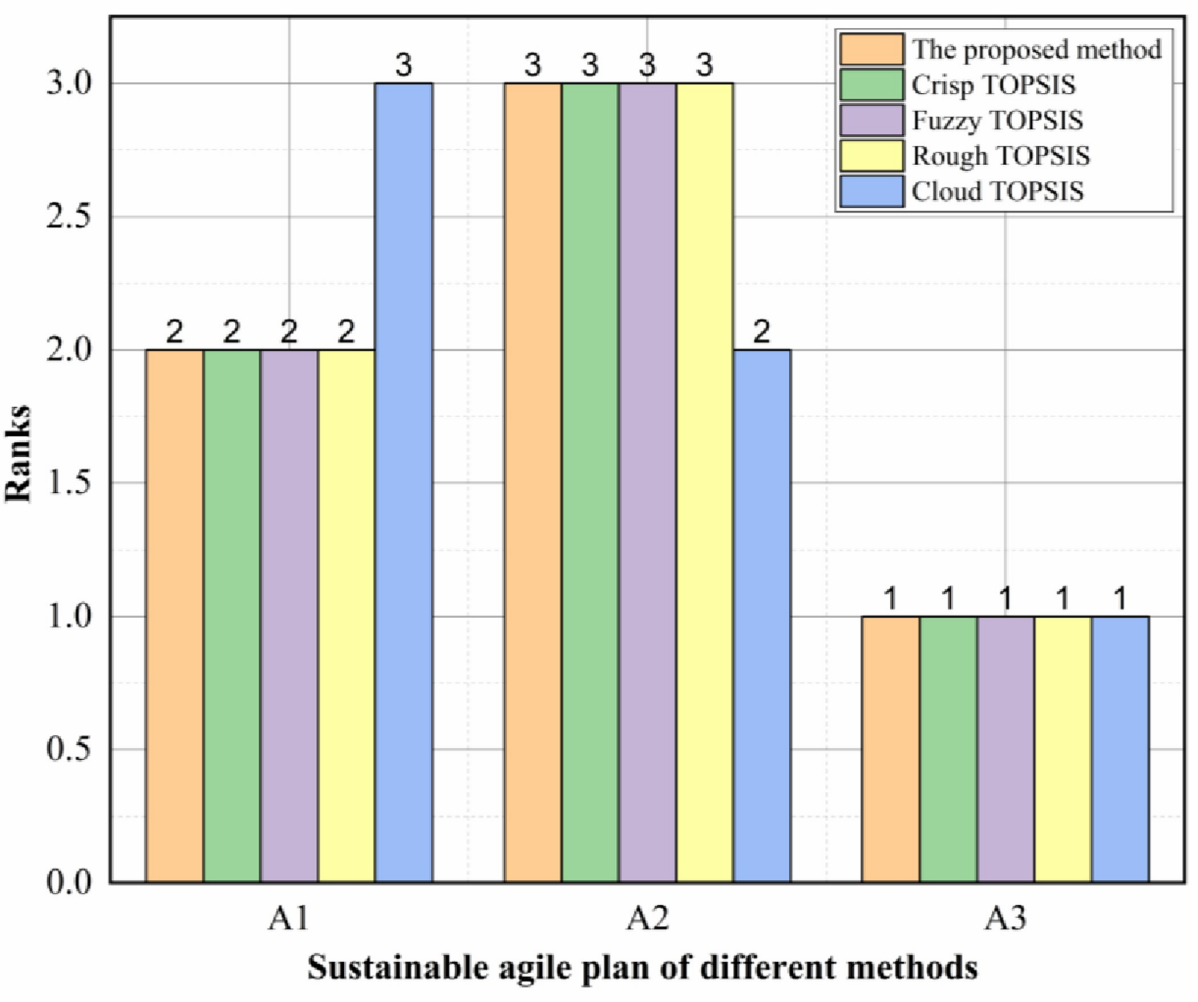
Ranking sustainable agile plans using various techniques.

**Fig 3:**
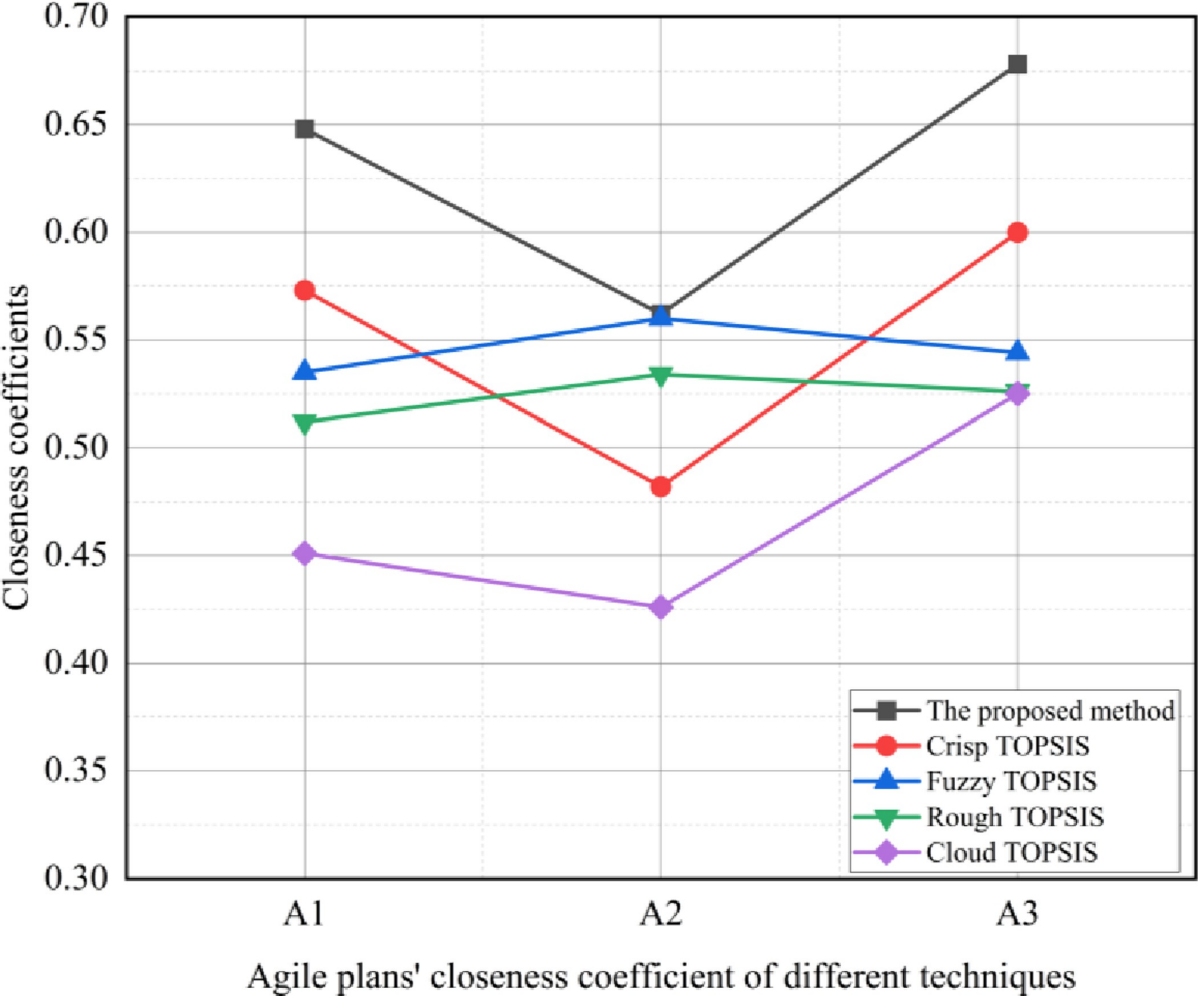
Closeness coefficient fof sustainable agile plans using different methods.

To assess the effectiveness and prevalence of the suggested approach, it was substituted with four widely recognized methods, which include crisp TOPSIS [64], cloud TOPSIS [65], rough TOPSIS [66], and fuzzy TOPSIS [67]. To ensure a fair comparison, all these methods were applied to the same case study involving China’s SMEs. The outcomes for sustainable agile plans using various techniques are shown in Figs 2 and 3. Fig 2 showcases the rankings of these sustainable agile plans using the various methods, where A3 secures the top rank based on the proposed method’s assessment, indicating it as the most sustainable plan among the options. Furthermore, Fig 3 demonstrates the closeness coefficients for each plan under different methods, with A3 consistently having the highest coefficient according to the proposed method’s evaluation.

This affirms that the proposed A3 plan surpasses the other techniques when it comes to ranking the most sustainable agile plan. In essence, the A3 method presents a distinct approach in assessing the sustainability of agile plans compared to the established methods. Consequently, it suggests that the proposed A3 method exhibits greater effectiveness for this specific application in evaluating sustainable plans for SMEs in China.

In future endeavors, this newly proposed technique will be implemented in a wider range of real-world decision-making scenarios to validate its broad applicability. By exploring hybrid approaches and modifying existing methodologies, researchers can address the limitations and leverage the strengths of current decision-making methods. This approach can lead to developing more robust, adaptable, and effective decision frameworks that better align with the complexities and requirements of real-world decision problems. Continuous experimentation, validation, and refinement are key to advancing the field of multi-criteria decision-making. Moreover, validating decision-making methods against real-world scenarios and diverse datasets is essential to establish their reliability, robustness, and suitability for practical use. Through systematic validation, analysis, and documentation, researchers can enhance confidence in the effectiveness and applicability of these methods, paving the way for their adoption in real decision-making contexts.

## 5. Conclusion

Sustainable agile product design has garnered growing attention within the contemporary business landscape. In this paper, a novel framework for selecting sustainable agile methods, based on SAPD practices, has been developed. This framework was applied in a case study involving the selection of sustainable agile product methods for China’s SMEs to assess its efficacy and practicality. The resulting ranking order for the case study on China’s SMEs is *A*3 > *A*1 > *A*2. The primary contributions of this study can be summarized as follows: Initially, SAPD practices are established as the criteria for evaluating the plans, encompassing economic, environmental, and social aspects. By scrutinizing the implementation of SAPD practices in the plans, potential markets with a focus on sustainability are identified and chosen. Within this context, the sustainable agile method is enhanced to prioritize cleanliness and sustainability. Secondly, to enhance the accuracy and realism of the rankings for sustainable agile plans, the method proposed here integrates the strengths of both cloud model theory and rough set theory. This combination effectively manages the various uncertainties present in the assessment process, especially regarding the importance of SAPD practices and the sustainability of markets under each practice. Cloud model theory is instrumental in accounting for the inherent randomness in judgment, while rough set theory addresses interpersonal uncertainty, all without the need for additional preset assumptions, as is often required in fuzzy set theory. In addition to employing interval linguistic terms, it’s important to note that experts often express uncertainty and hesitation in their initial judgments. Consequently, this approach considers various forms of uncertainty when assessing sustainable agile plans. Finally, a comprehensive integrated weighting method is utilized to calculate both subjective and objective weights, providing a holistic representation of the relative importance of SAPD practices.

In summary, the suggested approach offers several benefits as: 1) By employing cloud modeling to examine experts’ assessments’ randomness, the method addresses the limitation of fuzzy methodologies assuming crisp membership degrees. This enhances the accuracy of suppliers’ sustainability rankings. 2) Experts utilize interval linguistic terms to express preferences, reflecting uncertainty in linguistic choice. Unlike other methods, this approach acknowledges and incorporates this intrapersonal uncertainty, making it more reasonable. 3) Additionally, the proposed supplier selection method handles both intrapersonal and interpersonal uncertainties. Interpersonal uncertainty is managed through rough set theory, without requiring preset membership functions or fuzzy rules, resulting in a ranking more aligned with actual decisions. 4) Lastly, the approach incorporates an integrated weighting method that considers subjective and objective factors to determine the weights of SAPD practices. In contrast, other methods only consider subjective weights, making the new supplier selection method superior.

## Supporting information

S1 File Appendix (Tables A.1-A.7). (DOCX)
