# Supplementary material for: Sustainable agility of product development process based on a rough cloud technique: A case study on China’s small and medium enterprises
Source: PLoS One. 2024 Aug 22;19(8):e0300266. doi: 10.1371/journal.pone.0300266 (PMC11341065; doi:10.1371/journal.pone.0300266)
Supplement: S1 File — (DOCX) [file pone.0300266.s001.docx]

**S1 File.** **Appendix**

| Table A.1. ASDM practices for China’s SMEs Product Development (Sustainable design methods) | | |
| --- | --- | --- |
| Code Design Methods | | Description |
| Sd_1_ | Product Technology | Promote product innovation while maintaining adherence to technical standards [1]. |
| Sd_2_ | Design Methods | Selecting a well-designed hypothesis differs from scientific hypotheses because it involves innovative speculations and pre-structures, with the aim of achieving success [2]. |
| Sd_3_ | Product Material | It's essential to consider their financial limitations as well as their social and environmental consequences [3]. |
| Sd_4_ | Product Function | Facilitate individuals to partake in meaningful physical and mental pursuits while offering avenues for product interaction [4]. |
| Sd_5_ | Product Performance | Satisfy customer demands for product excellence, dependability, sustainability, and affordability, while also meeting businesses' needs for efficient product manufacturing and assembly [5]. |
| Sd_6_ | Market Demand | Market demand results from aggregating consumer preferences that aim to maximize their utility [6]. |
| Sd_7_ | Product Competition | In today's market, innovation, agility, and swiftness are imperative in the realm of new product development (NPD) within the industry. This is closely intertwined with the adoption of new manufacturing technologies, a vital component for achieving success and sustaining growth [7]. |
| Sd_8_ | Product Safety | The evaluation can be either qualitative or quantitative, depending on the product type and its intended use. These specifications are primarily dictated by legal requirements, regulations, and industry standards, or they may be specified by professional product customers, consumer organizations, or customer advocacy groups [8]. |
| Sd_9_ | Innovation Capability | The capacity to generate innovative and valuable products or knowledge, along with the capability to consistently convert knowledge and ideas into fresh products, processes, and systems that benefit the company and its stakeholders [9]. |
| Sd_10_ | Product Lifecycle | The complete progression of a product, from its readiness to enter the market to its eventual removal from the market, is referred to as the economic lifespan of the product or commodity within the market. It represents the lifecycle of the commodity, encompassing its journey from prosperity to decline [10]. |
| Sd_11_ | Organisation Management | The skill of uniting individuals on a shared platform to collaborate towards a predetermined common objective [11]. |
| Sd_12_ | Corporate Image | The public impression and how a business or brand is perceived by the public [12]. |
| Sd_13_ | Laws and Regulations | Guidelines and regulations established by government agencies to address behaviors that pose a threat to sustainable development [13]. |
| Sd_14_ | Production Capability | Enterprise capability is the capacity of a company to allocate resources effectively and compete. It stems from the amalgamation of tangible and intangible resources within the enterprise and reflects the performance level required for the company's daily production, operation, and management activities to support its development [14]. |
| Sd_15_ | Organization Decision | A sequence of strategic choices made by a company or one or more organizational units on behalf of the organization [15]. |
| Sd_16_ | Partner | Business partnerships entail a legal connection, and in the manufacturing sector, they represent a shared set of values. Sustainable value is established when shareholders and all other stakeholders jointly create value [16]. |
| Sd_17_ | Regional Policies | Regional policies refer to government measures aimed at boosting economic activities in particular regions within a country. The development of regional industrial ecology involves a three-stage process, starting with regional efficiency driven by independent decisions made by enterprises, progressing to regional learning characterized by mutual recognition, trust, and knowledge exchange, and ultimately evolving into sustainable industrial zones founded on the sustainable strategic vision of stakeholders [17]. |
| Sd_18_ | Efficient Operation and Production | Operational efficiency pertains to an organization's capacity to minimize time, energy, and material wastage while delivering high-quality services or products. Process optimization aims to achieve maximum productivity within given constraints [18]. |
| Sd_19_ | Social Culture | Social culture represents shared traditions, customs, behaviors, and beliefs within a group, and it plays a paramount role in shaping individuals' decisions within society. The promotion of stakeholder sustainability is guaranteed by crucial social and cultural components, including equity, consciousness, engagement, and unity [19]. |
| Sd_20_ | Network Relationship | It's the linkage between pertinent business entities that facilitates companies to cooperate in enhancing their expertise, knowledge, and specialized skills [20]. |
| Sd_21_ | Product Comprehensive Service | It involves a blend of offerings provided by a company to fulfil customer requirements. Products are physical items, whereas services are intangible actions. In contrast to the design of physical products, product service design places greater emphasis on the value derived from use [21]. |
| Sd_22_ | Information and Knowledge Acquisition | Knowledge and information acquisition involves the procedures of obtaining, processing, comprehending, and remembering information and experiences. It's an interactive process, and a diverse range of participants enhances the innovation process [22]. |
| Sd_23_ | Employee Ability Level | It refers to an individual's expertise in a particular subject. Enterprises aim to encourage their employees to be more productive and efficient, work creatively, and generate diverse ideas, considering their skill levels [23]. |
| Sd_24_ | Manager Preference | Management preferences encompass the inclinations, aversions, convictions, and viewpoints of managers regarding their involvement in the strategic planning process. Managers may develop personal interests that could lead them to take actions that are detrimental to sustainable development [24-25]. |

| Table A.2. ASDM practices for China’s SMEs Product Development (Agile management methods) | | |
| --- | --- | --- |
| Methods | | Description |
| Am_1_ | Flexible Process | Enabling businesses to swiftly adapt their operational procedures in response to shifts in their surroundings [26]. |
| Am_2_ | Time-based Manufacturing | This practice centers on catering to the needs and preferences of customers. It encompasses various strategies such as cellular manufacturing, reengineering setups, quality enhancement initiatives, preventive maintenance, and pull production. It fosters a corporate culture that emphasizes collaboration with colleagues, eschews authoritarian management approaches, promotes integration with suppliers, and advocates investments in infrastructure and equipment [27]. |
| Am_3_ | Continuous Manufacturing | A coordinated arrangement of unit operations, characterized by continuous flow, whether steady or periodic and employing control systems based on models [27]. |
| Am_4_ | Build to order Production | Empowering the company to tailor the product to meet the specific needs of its customers [27]. |
| Am_5_ | Efficient System | Separating the creation of economic value from the consumption of materials and energy [28]. |
| Am_6_ | Fulfilment and Diversified of Demand | This myopic re-optimization of products pertains to addressing a variety of recently accepted requests. It can be accomplished by utilizing initially created (meta) heuristics for solving static issues or by employing newly designed routing algorithms [29]. |
| Am_7_ | Customization of Production | To accommodate specific requirements of individuals, it is often necessary to reorganize service processes and allocate resources differently. In some cases, a complete redesign of the entire production process may be required to adapt to these changes [21]. |
| Am_8_ | Integrated Innovation | Providing startup opportunities to create and provide environmentally friendly and marketable products, in alignment with the Open Sustainable Innovation paradigm [30]. |

| Table A.3. Linguistic term scale | |
| --- | --- |
| Linguistic term (S) | The importance |
| S_0_ | Very poor |
| S_1_ | Poor |
| S_2_ | Fair |
| S_3_ | Good |
| S_4_ | Very good |

| Table A.4. The associated interval linguistic descriptions and numerical feature values in the cloud model [31]. | | | | | |
| --- | --- | --- | --- | --- | --- |
| Level | Very poor | Poor | Fair | Good | Very good |
| Scope | 0-0.25 | 0-0.48 | 0.27-0.75 | 0.51-0.99 | 0.76-1.00 |
| Ex | 0.001 | 0.245 | 0.505 | 0.755 | 1.000 |
| En | 0.060 | 0.060 | 0.060 | 0.060 | 0.060 |
| He | 0.002 | 0.005 | 0.005 | 0.005 | 0.002 |

| Table A.5. Linguistic term scale for assessment of SAPD practices and product development plans | | |
| --- | --- | --- |
| Linguistic term | Relative importance of SAPD practices | Performance of product development plans |
| S_0_ | Very poor | Very low |
| S_1_ | Poor | low |
| S_2_ | Fair | Fair |
| S_3_ | Good | High |
| S_4_ | Very good | Very high |

| Table A.6. Experts’ preferences for the SAPD practices. | | | | | | | | | | | | | | | | | | |
| --- | --- | --- | --- | --- | --- | --- | --- | --- | --- | --- | --- | --- | --- | --- | --- | --- | --- | --- |
| Practices | | | | $k_{1}$ | | | $k_{2}$ | | | $k_{3}$ | | | $\cdots$ | | | $k_{10}$ | | |
| Sd_1_ | | | | G-G | | | G-G | | | M-M | | | $\cdots$ | | | G-VG | | |
| Sd_2_ | | | | VG-VG | | | G-VG | | | M-G | | | $\cdots$ | | | G-G | | |
| Sd_3_ | | | | VG-VG | | | VG-VG | | | VG-VG | | | $\cdots$ | | | VG-VG | | |
| Sd_4_ | | | | G-VG | | | G-G | | | G-G | | | $\cdots$ | | | VG-VG | | |
| Sd_5_ | | | | M-M | | | G-G | | | M-M | | | $\cdots$ | | | G-G | | |
| Sd_6_ | | | | M-G | | | G-VG | | | G-VG | | | $\cdots$ | | | G-G | | |
| Sd_7_ | | | | VG-VG | | | VG-VG | | | G-VG | | | $\cdots$ | | | VG-VG | | |
| Sd_8_ | | | | P-M | | | M-M | | | P-P | | | $\cdots$ | | | VP-P | | |
| Sd_9_ | | | | VP-VP | | | VP-P | | | VP-VP | | | $\cdots$ | | | VP-VP | | |
| Sd_10_ | | | | M-M | | | P-M | | | M-M | | | $\cdots$ | | | M-M | | |
| Sd_11_ | | | | M-G | | | M-M | | | P-M | | | $\cdots$ | | | P-P | | |
| Sd_12_ | | | | M-M | | | M-M | | | M-M | | | $\cdots$ | | | P-M | | |
| Sd_13_ | | | | G-G | | | VG-VG | | | G-VG | | | $\cdots$ | | | G-G | | |
| Sd_14_ | | | | M-G | | | M-M | | | G-G | | | $\cdots$ | | | M-G | | |
| Sd_15_ | | | | G-G | | | VG-VG | | | G-VG | | | $\cdots$ | | | M-G | | |
| Sd_16_ | | | | M-G | | | G-G | | | M-G | | | $\cdots$ | | | G-G | | |
| Sd_17_ | | | | G-VG | | | G-G | | | M-G | | | $\cdots$ | | | M-G | | |
| Sd_18_ | | | | M-G | | | M-G | | | G-G | | | $\cdots$ | | | P-P | | |
| Sd_19_ | | | | G-VG | | | G-G | | | M-G | | | $\cdots$ | | | P-M | | |
| Sd_20_ | | | | G-G | | | G-G | | | G-G | | | $\cdots$ | | | VG-VG | | |
| Sd_21_ | | | | G-G | | | G-VG | | | VG-VG | | | $\cdots$ | | | P-P | | |
| Sd_22_ | | | | G-G | | | G-G | | | M-G | | | $\cdots$ | | | M-M | | |
| Sd_23_ | | | | G-G | | | M-G | | | M-M | | | $\cdots$ | | | G-G | | |
| Sd_24_ | | | | M-G | | | G-G | | | G-G | | | $\cdots$ | | | G-G | | |
| Am_1_ | | | | M-M | | | G-G | | | M-M | | | $\cdots$ | | | P-M | | |
| Am_2_ | | | | G-G | | | M-G | | | M-M | | | $\cdots$ | | | P-P | | |
| Am_3_ | | | | VG-VG | | | VG-VG | | | G-VG | | | $\cdots$ | | | VG-VG | | |
| Am_4_ | | | | VG-VG | | | VG-VG | | | G-VG | | | $\cdots$ | | | G-G | | |
| Am_5_ | | | | M-G | | | G-G | | | M-G | | | $\cdots$ | | | G-G | | |
| Am_6_ | | | | M-G | | | M-G | | | G-VG | | | $\cdots$ | | | G-G | | |
| Am_7_ | | | | P-P | | | P-M | | | M-G | | | $\cdots$ | | | M-M | | |
| Am_8_ | | | | P-P | | | P-P | | | M-M | | | $\cdots$ | | | P-P | | |
| Table A.7. Experts’ ratings of the three design plans | | | | | | | | | | | | | | | | | | |
| Practices | $k_{1}$ | | | | $k_{2}$ | | | | $k_{3}$ | | | | |  | $k_{10}$ | | | |
|  | $A1$ | $A2$ | $A3$ | | $A1$ | $A2$ | | $A3$ | $A1$ | | $A2$ | $A3$ | | $\cdots$ | $A1$ | | $A2$ | $A3$ |
| Sd_1_ | VL-VL | VL-L | F-H | | VL-VL | L-L | | H-H | L-L | | F-F | H-H | | $\cdots$ | VL-VL | | F-F | VH-VH |
| Sd_2_ | H-H | H-H | VH-VH | | H-H | H-H | | H-H | F-H | | F-F | F-F | | $\cdots$ | H-H | | H-H | VH-VH |
| Sd_3_ | F-H | F-H | F-F | | F-F | F-F | | H-H | F-H | | F-H | H-H | | $\cdots$ | VH-VH | | VH-VH | VH-VH |
| Sd_4_ | H-H | H-H | VH-VH | | F-H | F-H | | H-H | F-H | | F-H | H-H | | $\cdots$ | L-L | | F-H | VL-VL |
| Sd_5_ | F-F | H-H | L-L | | F-F | F-F | | L-L | H-H | | VH-VH | F-H | | $\cdots$ | H-H | | H-H | H-H |
| Sd_6_ | H-H | F-H | F-F | | H-H | H-H | | L-L | H-H | | VH-VH | L-L | | $\cdots$ | H-H | | F-H | F-F |
| Sd_7_ | F-H | H-H | VH-VH | | H-H | H-H | | F-H | F-F | | F-H | L-F | | $\cdots$ | VH-VH | | VH-VH | H-H |
| Sd_8_ | VH-VH | VH-VH | F-H | | VH-VH | VH-VH | | L-F | H-H | | H-H | F-F | | $\cdots$ | H-VH | | H-VH | VH-VH |
| Sd_9_ | H-H | H-H | VH-VH | | H-H | H-H | | H-H | F-H | | L-F | H-H | | $\cdots$ | H-H | | H-H | VH-VH |
| Sd_10_ | H-H | H-H | VL-VL | | VH-VH | VH-VH | | VL-L | H-H | | H-H | L-L | | $\cdots$ | H-H | | H-H | L-L |
| Sd_11_ | F-F | F-H | VH-VH | | H-H | H-H | | VH-VH | VH-VH | | F-F | H-H | | $\cdots$ | H-H | | F-H | H-H |
| Sd_12_ | VH-VH | VH-VH | VH-VH | | H-H | VH-VH | | VH-VH | H-VH | | VH-VH | VH-VH | | $\cdots$ | VL-VL | | F-H | H-H |
| Sd_13_ | L-L | L-L | L-F | | H-H | H-H | | H-H | F-F | | F-H | F-H | | $\cdots$ | H-VH | | H-VH | H-VH |
| Sd_14_ | H-H | H-H | H-H | | H-VH | H-VH | | H-VH | F-H | | F-F | F-F | | $\cdots$ | VH-VH | | H-H | H-H |
| Sd_15_ | VH-VH | VH-VH | L-L | | H-VH | H-H | | L-F | H-H | | H-H | F-F | | $\cdots$ | VH-VH | | VH-VH | VH-VH |
| Sd_16_ | H-H | F-H | VL-L | | H-H | F-F | | L-L | VH-VH | | H-H | H-H | | $\cdots$ | H-VH | | H-VH | H-VH |
| Sd_17_ | H-H | H-H | H-H | | VH-VH | VH-VH | | VH-VH | F-H | | H-H | VH-VH | | $\cdots$ | F-H | | F-H | F-H |
| Sd_18_ | H-H | H-H | L-F | | F-F | F-F | | VL-VL | H-H | | H-VH | L-L | | $\cdots$ | H-H | | H-H | H-H |
| Sd_19_ | H-H | H-H | H-H | | F-H | F-H | | F-H | VH-VH | | VH-VH | VH-VH | | $\cdots$ | H-H | | H-H | H-H |
| Sd_20_ | F-H | F-H | F-H | | H-H | H-H | | H-H | H-H | | F-F | F-F | | $\cdots$ | F-F | | F-F | H-H |
| Sd_21_ | F-F | F-F | H-H | | L-L | L-L | | H-H | L-L | | L-L | H-VH | | $\cdots$ | H-H | | H-H | H-VH |
| Sd_22_ | H-VH | H-VH | VH-VH | | H-H | H-H | | H-VH | H-VH | | VH-VH | VH-VH | |  | H-VH | | H-VH | H-VH |
| Sd_23_ | H-VH | H-VH | VH-VH | | VH-VH | VH-VH | | VH-VH | H-H | | H-H | H-H | |  | F-F | | F-F | F-F |
| Sd_24_ | H-H | H-H | H-H | | H-H | H-H | | H-H | VH-VH | | VH-VH | VH-VH | |  | H-H | | H-H | H-H |
| Am_1_ | F-H | H-H | H-H | | L-F | L-F | | L-L | F-F | | F-H | L-L | | $\cdots$ | H-H | | H-H | H-H |
| Am_2_ | H-H | H-H | H-H | | H-VH | H-VH | | H-VH | VH-VH | | VH-VH | VH-VH | | $\cdots$ | H-H | | H-H | H-H |
| Am_3_ | L-L | L-F | L-L | | H-H | H-H | | L-L | VH-VH | | VH-VH | VL-VL | | $\cdots$ | H-H | | H-H | F-F |
| Am_4_ | H-VH | H-VH | H-VH | | H-H | H-H | | H-H | VH-VH | | VH-VH | VH-VH | | $\cdots$ | F-H | | F-H | F-H |
| Am_5_ | F-H | F-H | F-H | | H-H | H-H | | H-H | F-F | | F-F | F-F | | $\cdots$ | L-F | | L-F | L-F |
| Am_6_ | H-H | VH-VH | VH-VH | | H-H | VH-VH | | F-H | VH-VH | | H-H | F-H | | $\cdots$ | H-H | | VH-VH | H-H |
| Am_7_ | L-L | L-L | H-H | | VL-VL | VL-VL | | F-H | VL-L | | VL-L | L-F | | $\cdots$ | F-H | | F-H | F-H |
| Am_8_ | H-H | H-H | H-VH | | F-F | F-H | | H-VH | F-H | | F-H | H-VH | | $\cdots$ | H-H | | H-H | H-VH |

**Reference**

1. Li, G., Wang, X., Su, S., & Su, Y. (2019). How green technological innovation ability influences enterprise competitiveness. *Technology in Society, 59,* 101136.
2. Hall, W., & Keynes, M. (1981). Design method and scientific method. *Design Studies*, *2*(4), 195–201.
3. Arroyo, P. of C. E. and M., Tommelein, I. D., & Ballard, G. (2016). Selecting globally sustainable materials: A case study using choosing by advantages. *Journal of Construction Engineering and Management*. https://ascelibrary.org/doi/abs/10.1061/(ASCE)CO.1943-7862.0001041
4. Ratneshwar, S. (2001). *Innovations in Product Functionality : When and Why Are Explicit Comparisons Effective ?* 49–61.
5. Nguyen, D. S., Vignat, F., & Brissaud, D. (2010). *Product performance simulation with geometric deviations throughout its life cycle: Vol. 66 AISC* (pp. 79–95). Springer Verlag. https://doi.org/10.1007/978-3-642-10430-5_7
6. Lahiri, S. (2020). Consumer surplus and budget constrained preference maximization: A note. *Managerial Economics, 21(1)*, 49-65.
7. De Almeida, J. F., Amaral, D. C., & Coelho, R. T. (2021). Innovative Framework to manage New Product Development (NPD) Integrating Additive Manufacturing (AM) and Agile Management. *Procedia CIRP*, *103*, 128–133. https://doi.org/10.1016/j.procir.2021.10.020
8. Rausand, M., & Utne, I. B. (2009). Product safety – Principles and practices in a life cycle perspective. *Safety Science*, *47*(7), 939–947. https://doi.org/10.1016/j.ssci.2008.10.004
9. Saunila, M. (2019). Innovation capability in SMEs: A systematic review of the literature. *Journal of Innovation & Knowledge*, 2–7. https://doi.org/10.1016/j.jik.2019.11.002
10. Maropoulos, P. G., & Ceglarek, D. (2010). CIRP Annals - Manufacturing Technology Design verification and validation in product lifecycle. *CIRP Annals - Manufacturing Technology*, *59*, 740–759. https://doi.org/10.1016/j.cirp.2010.05.005
11. Nanthasudsawaeng, K. (2023). Systematical Development to Personnel Efficient Teamwork in King Mongkut ’ s University of Technology North Bangkok Rayong Campus. *Journal of Multidisciplinary in Humanities and Social Sciences*.
12. Zinkhan, M. J., & M., G. (1984). DEFINING AND MEASURING COMPANY IMAGE. *Developments in Marketing Science: Proceedings of the Academy of Marketing Science*, 346–350.
13. Omri, A., & Ben, N. (2020). Good governance for sustainable development goals : Getting ahead of the pack or falling behind ? *Environmental Impact Assessment Review*, *83*(March), 106388. https://doi.org/10.1016/j.eiar.2020.106388
14. Vanegas, J. A., & Pearce, A. R. (2016). Sustainable Design and Construction Strategies for the Built Environment. *Researchgate.Net*, *June*.
15. James, H. S., & James, S. (2014). *Reinforcing Making Organizational Ethical Decision Structure*. *28*(1), 43–58.
16. Badurdeen, F. for S. M., & Jawahir, I. (2017). Strategies for value creation through sustainable manufacturing. *ScienceDirect*.
17. Zhu, J., & Ruth, M. (2014). The development of regional collaboration for resource efficiency: A network perspective on industrial symbiosis. *Computers, Environment and Urban Systems*, *44*, 37–46. https://doi.org/10.1016/j.compenvurbsys.2013.11.001
18. Tripathi, V., Chattopadhyaya, S., Mukhopadhyay, A. K., Sharma, S., Singh, J., Pimenov, D. Y., & Giasin, K. (2021). An innovative agile model of smart lean–green approach for sustainability enhancement in industry 4.0. *Journal of Open Innovation: Technology, Market, and Complexity*, *7*(4), 215. https://doi.org/10.3390/joitmc7040215
19. Maskil-Leitan, R., & Reychav, I. (2018). A sustainable sociocultural combination of building information modeling with integrated project delivery in a social network perspective. *Clean Technologies and Environmental Policy*, *20*(5), 1017–1032.
20. Huang, C., & Wang, Y. (2018). Evolution of network relations, enterprise learning, and cluster innovation networks: the case of the Yuyao plastics industry cluster. *Technology Analysis and Strategic Management*, *30*(2), 158–171. https://doi.org/10.1080/09537325.2017.1297786
21. Song, W., & Sakao, T. (2017). A customization-oriented framework for design of sustainable product/service system. *Journal of Cleaner Production*.
22. Yao, C.-Y. (2012). *Knowledge diversity, knowledge interaction, organizational climate and business innovation*. 129–134. https://doi.org/10.1109/ICMIT.2012.6225792
23. Rahimi, Z., Resi, E., & Kožo, A. (2012). *Determining the Level of Management Competences in the Process of Employee Motivation*. *41*, 535–543. https://doi.org/10.1016/j.sbspro.2012.04.066
24. Chao, C. (1996). Managers ’ Preferences and Trade-offs in Supplier Selection and Performance Evaluation Venkatapparao Mummalaneni. *Industrial Marketing Management*, *124*(1996), 115–124.
25. Gormley, T. A., & Matsa, D. A. (2016). Playing it safe? Managerial preferences, risk, and agency conflicts. *SSRN Electronic Journal*.
26. Schonenberg, H., Weber, B., & Dongen, B. Van. (2008). *Supporting Flexible Processes through Recommendations Based on History*. 51–66.
27. Nahm, A. Y., & Vonderembse, M. A. (2004). *The Impact of Organizational Culture on Time-Based Manufacturing and Performance*. *35*(4), 579–607.
28. Ceschin, F. of E. & D. (2013). Critical factors for implementing and diffusing sustainable product-Service systems: Insights from innovation studies and companies’ experiences. *Journal of Cleaner Production*, *45*, 74–88. https://doi.org/10.1016/j.jclepro.2012.05.034
29. Haferkamp, J., & Ehmke, J. F. (2021). *Effectiveness of demand and fulfillment control in dynamic fleet management of ride-sharing systems*. *April*, 1–24. https://doi.org/10.1002/net.22062
30. Cappa, F. of B. and M., Sette, F. Del, Hayes, D., Rosso, F., Asdrubali, F., & Buzzini, P. (2016). How to Deliver Open Sustainable Innovation: An Integrated Approach for a Sustainable Marketable Product. *Sustainability Article*. https://doi.org/10.3390/su8121341
31. Liu, H. C., Li, Z., Song, W., & Su, Q. (2017). Failure mode and effect analysis using cloud model theory and PROMETHEE method. *IEEE Transactions on Reliability, 66(4)*, 1058-1072.
